# Unravelling the Role of *O*-glycans in Influenza A Virus Infection

**DOI:** 10.1038/s41598-018-34175-3

**Published:** 2018-11-06

**Authors:** Juliane Mayr, Kam Lau, Jimmy C. C. Lai, Ivan A. Gagarinov, Yun Shi, Sarah McAtamney, Renee W. Y. Chan, John Nicholls, Mark von Itzstein, Thomas Haselhorst

**Affiliations:** 1 Institute for Glycomics, Gold Coast Campus, Griffith University, Queensland, 4222 Australia; 2 Department of Pathology, University of Hong Kong, Hong Kong, SAR China; 3 HKU-Pasteur Research Centre, Hong Kong, SAR China; 4 Department of Paediatrics, Faculty of Medicine, The Chinese University of Hong, Hong Kong, SAR China

## Abstract

The initial stage of host cell infection by influenza A viruses (IAV) is mediated through interaction of the viral haemagglutinin (HA) with cell surface glycans. The binding requirement of IAVs for Galβ(1,4)Glc/ GlcNAc (lactose/lactosamine) glycans with a terminal α(2,6)-linked (human receptors) or α(2,3)-linked (avian receptors) *N*-acetylneuraminic residue commonly found on *N*-glycans, is well-established. However the role and significance of sialylated Galβ(1,3)GalNAc (core 1) epitopes that are typical *O*-glycoforms in influenza virus pathogenesis remains poorly detailed. Here we report a multidisciplinary study using NMR spectroscopy, virus neutralization assays and molecular modelling, into the potential for IAV to engage sialyl-Galβ(1,3)GalNAc *O*-glycoforms for cell attachment. H5 containing virus like particles (VLPs) derived from an H5N1 avian IAV strain show a significant involvement of the *O*-glycan-specific GalNAc residue, coordinated by a EQTKLY motif conserved in highly pathogenic avian influenza (HPAI) strains. Notably, human pandemic H1N1 influenza viruses shift the preference from ‘human-like’ α(2,6)-linkages in sialylated Galβ(1,4)Glc/GlcNAc fragments to ‘avian-like’ α(2,3)-linkages in sialylated Galβ(1,3)GalNAc without involvement of the GalNAc residue. Overall, our study suggests that sialylated Galβ(1,3)GalNAc as *O*-glycan core 1 glycoforms are involved in the influenza A virus life cycle and play a particularly crucial role during infection of HPAI strains.

## Introduction

**Figure 1:**
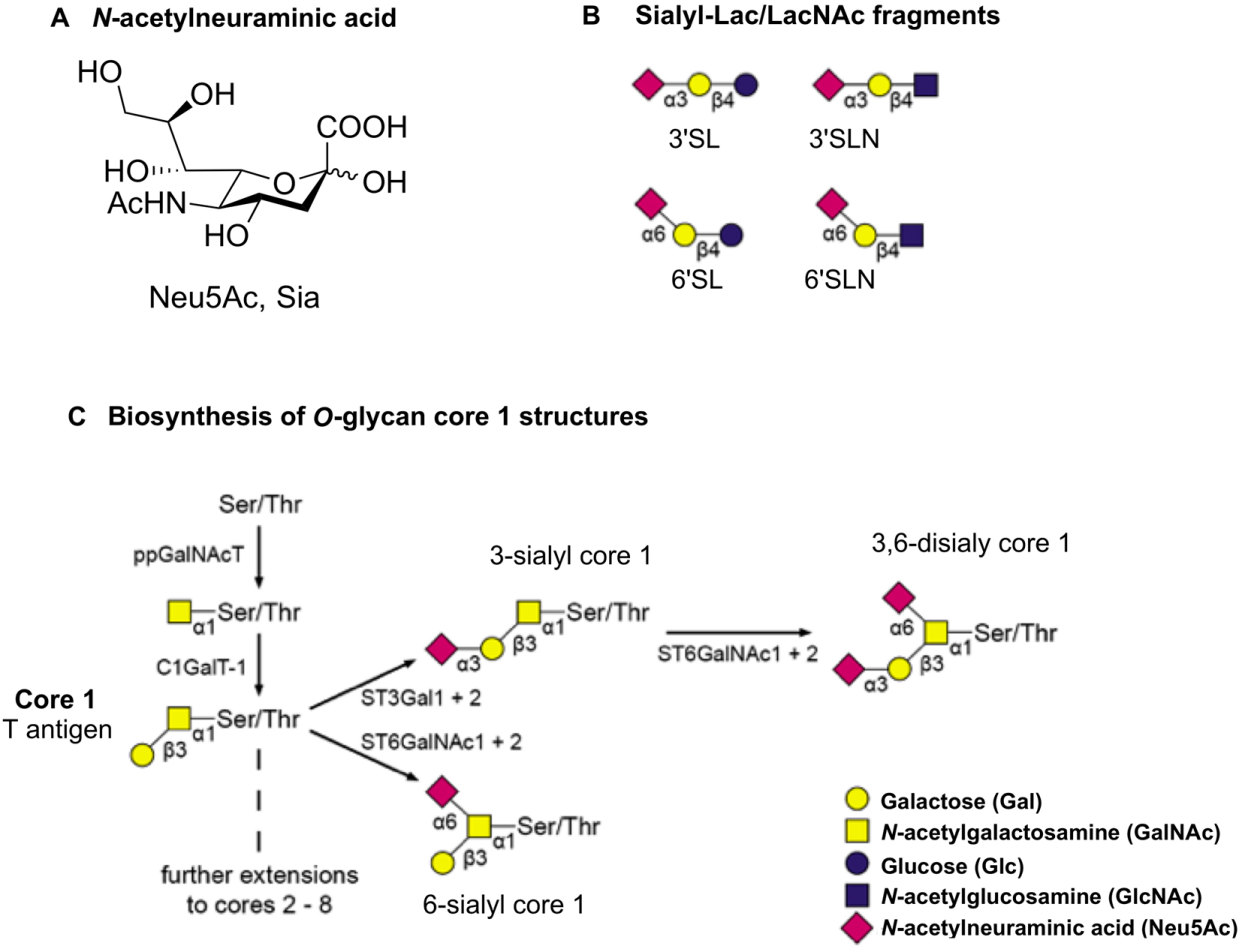
(**A**) *N*-acetylneuraminic acid (Neu5Ac, sialic acid (Sia)). (**B**) Standard receptor analogues Sialyl-Lac/LacNAc fragments: α(2,3)-sialyllactose [3′SL, Neu5Acα(2,3)Galβ(1,4)Glc], α(2,3)-sialyllactosamine (3′SLN, Neu5Acα(2,3)Galβ(1,4)GlcNAc]), α(2,6)-sialyllactose [6′SL, Neu5Acα(2,6)Galβ(1,4)Glc], α(2,6)-sialyllactosamine (6′SLN, Neu5Acα(2,6)Galβ(1,4)GlcNAc]). (**C**) Overview of the biosynthesis of sialylated core 1 *O*-glycans: The *O*-glycosylation is initiated by linking GalNAc to a serine or threonine via polypeptide-*N*-acetylgalactosaminyl-transferases (ppGalNAcTs). Core 1 β(1,3)galactosyltransferase (C1GalT1) adds a Gal residue to form the core 1 [T antigen, Galβ(1,3)GalNAcα-Ser/Thr]^45^. Core 1 can be substituted with Neu5Ac catalyzed by ST3 β-galactoside α(2,3)-sialyltransferase 1 and 2 (ST3Gal1 and 2) forming Neu5Acα(2,3)Galβ(1,3)GalNAcα-Ser/Thr (3-sialyl core 1)^46^. ST6 α-*N*-acetyl-neuraminyl-(2,3)-β-galactosyl-1,3-*N*-acetylgalactose-aminide-α(2,6)-sialyltransferase 1 and 2 (ST6GalNAc1 and 2) catalyze the formation of the disialylated core 1 [Neu5Acα(2,6)[Neu5Acα(2,3)Galβ(1,3)]GalNAcα-Ser/Thr; 3,6-disialyl core 1] and Neu5Acα(2,6)[Galβ(1,3)]GalNAcα-Ser/Thr (6-sialyl core 1)^47,48^.

It is commonly accepted that IAV host cell tropism and transmission is based on the nature of the *N*-acetylneuraminic acid (Neu5Ac) linkage to the penultimate Gal residue of cell surface glycan receptors (Fig. 1A,B), however, it contributes only partially to the understanding of the complex interactions of glycan receptors and influenza A viruses. Avian HAs commonly prefer to bind Neu5Ac α(2,3)-linked to Gal but distinguish between the structures of the inner residues of the oligosaccharides^1^. It has also been shown that HA binds to 6′-*O*-sulfated-*N*-acetyllactosamine [6′-HSO_3_-Galβ(1,4)GlcNAc] with similar affinity as to α(2,6)-sialyllactose [6′SL, Neu5Acα(2,6)Galβ(1,4)Glc]^2^. Several human and avian virus HAs, including that from the 2009 pandemic H1N1, show binding to glycans with sulfation, fucosylation and internal sialylation^3,4^. It has also been suggested that the structural topology of the sialyloligosaccharide [*cone-like* for α(2,3)-sialylated or *umbrella-like* for α(2,6)-sialylated glycans], rather than the Neu5Ac glycosidic linkage itself, is essential for binding specificity of HA to α(2,6)-sialylated glycans^5^.

Most HA receptor specificity studies to date have investigated extensively the role of Sialyl-Galβ(1,4)Glc/GlcNAc (=Sialyl-Lac/LacNAc) fragments, but the involvement of *O*-glycan specific Sialyl-Galβ(1–3)GalNAc sequences in the influenza virus-host interactions is poorly understood. It has been reported that IAV interacts with branched sialylated *O*-glycoforms, such as disialylated core 1 *O*-glycan, on natural killer receptor NKp46^6^. Furthermore, in ferrets human-adapted IAV utilizes α(2,6)-sialylated *O*-glycans^7^. The most common *O*-glycosylation in higher eukaryotes is mucin-type *O*-glycosylation^8^, in which *N*-acetylgalactosamine (GalNAc) is α-glycosidically linked to the hydroxyl group of serine or threonine (Fig. 1C)^9^. So far, eight different *O*-GalNAc glycan core structures have been described: core structures 1 and 2 are found in both glycoproteins and mucins from a range of cells and tissues whereas *O*-glycan cores 3–8 have a more restricted occurrence^8^. Mono- and disialylated core 1 *O*-glycans were detected in respiratory tract tissues of ferrets and as well in human lung and bronchus tissues^10,11^.

## Results

Here, we report the role of Sialyl-Galβ(1–3)GalNAc in IAV infection at a molecular and atomic level by a multidisciplinary experimental approach. Significant variations between virus strains were detected including the importance of a non-sialic acid glycan residue. This study provides more detailed insights into the complexity of influenza virus-host-interactions and suggests that Sialyl-Lac/LacNAc are not the only influenza virus receptors. First, a histochemical analysis using antibodies to evaluate the expression of sialyltransferases (ST), especially ST6GalNAc1 and ST6GalNAc2 that are essential in the biosynthesis of core 1 *O*-linked glycans (Fig. 1C)^11,12^, was carried out. Both, type 1-like pneumocytes and A549 human lung cell lines, showed strong expression of ST6GalNAc1 but little ST6Gal1 or ST6GalNAc2 expression (Supplementary Fig. 1A), indicating that binding of human IAV would preferentially use Sialyl-*O*-glycan motifs such as 6-sialyl-Galβ(1–3)GalNAc and 3,6-disialyl-Galβ(1–3)GalNAc. The low expression of ST6Gal1, predominantly involved in the biosynthesis of sialylated *N*-glycans, suggests that *O*-glycans might play a role in IAV cell recognition. Immunohistochemical analysis of normal adult lung and bronchial tissues also showed an elevated expression of ST6GalNAc1 in bronchus and bronchioles, with ST6Gal1 expression in the bronchial epithelium. Upper respiratory nasopharyngeal tissue staining showed positive punctate staining for ST6GalNAc1 and ST6Gal1 in the cytoplasm while ST6GalNAc2 was mainly confined to infiltrating inflammatory cells (Supplementary Fig. 1B).

### Chemoenzymatic synthesis delivers Sialyl-Galβ(1,3)GalNAc

**Figure 2:**
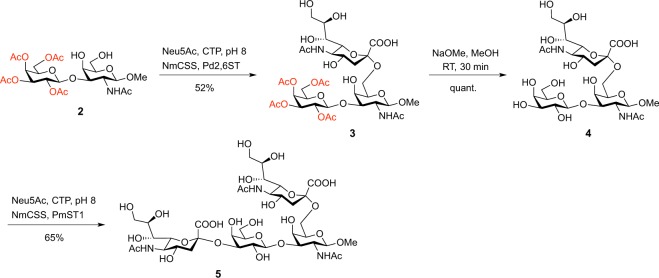
Chemoenzymatic synthesis of sialyl-Galβ(1,3)GalNAc. Acetate protection allows the regioselective incorporation of α(2,6)-linked *N*-acetylneuraminic acid. See Supplementary Information for further details.

To evaluate the glycointeractome of Sialyl-Galβ(1–3)GalNAc and IAVs we have developed a chemoenzymatic approach for the synthesis of Sialyl-Galβ(1–3)GalNAc and its derivatives^13–16^. We suggest using a single Galβ(1,3)GalNAc disaccharide precursor, from which the target 6-sialylated core 1 tri- and tetrasaccharides are synthesized. To generate the 6-sialylated core 1 structure, the hydroxyl groups of the terminal Gal residue were protected from unwanted sialic acid transfer with acetyl (Ac) groups (2, Fig. 2). This allowed absolute regioselective introduction of Neu5Ac onto C6 of the GalNAc residue using a commercially available α(2,6)-sialyltransferase (Pd2,6ST) (3, Fig. 2). The acetyl esters on the Gal residue were then removed quantitatively with a catalytic amount of sodium methoxide in methanol to produce the 6-sialylated core 1 derivative, Galβ(1,3)-[Neu5Acα(2,6)]-GalNAcβOMe (4), with the Gal moiety unmasked for further enzymatic transformation. Subsequent sialylation of 4 with α(2,3)-sialyltransferase (PmST1) gave the 3,6-disialylated tetrasaccharide as the β-methyl glycoside derivative, Neu5Acα(2,3)-Galβ(1,3)-[Neu5Ac-α(2,6)]-GalNAcβOMe (5). We have also synthesised sialylated *O*-glycans as an α-serine glycoside derivative (6) that resembles the biologically relevant configuration following published methods^17^.

### Recognition of Sialyl-Lac/LacNAc fragments by influenza virus HA studied by STD NMR spectroscopy

The binding of four Sialyl-Lac and Sialyl-LacNAc trisaccharides (3′SL, 6′SL, 3′SLN, and 6′SLN; Fig. 1B) to intact influenza virus particles [A/Hong Kong/1870/2008(H1N1) (H1N1sea), A/Perth/16/2009(H3N2) (H3N2), and A/California/04/2009(H1N1) (H1N1pdm)] was then studied by Saturation Transfer Difference (STD) NMR spectroscopy (Supplementary Fig. 2 and 3). We and others have studied the interaction of ligands with whole virions and virus like particles (VLPs) by NMR spectroscopy^18–23^. The most abundant protein on the IAV surface is HA making up ~80% of the surface expressed viral proteins, followed by the neuraminidase (NA) at ~17% and matrix protein with approximately 16–20 molecules^24^. To prevent binding of the receptor analogues to the NA and consequently to block receptor-destroying activity, NA-specific inhibitor oseltamivir carboxylate (OC) was added at low concentrations without interference with the NMR analysis^19,25^. All investigated IAV strains were able to bind 3′SL and 6′SL but with strain-specific differences (Supplementary Fig. 2). We have previously reported that H5-VLPs of H5N1 origin preferred avian-like 3′SL over 6′SL^23^. In contrast, the NMR signals of 3′SL and 6′SL in complex with H3N2 were of similar intensities (Supplementary Fig. 2b), suggesting a comparable binding affinity to α(2,6)- and α(2,3)-linked Neu5Ac. H1N1pdm and H1N1sea on the other hand clearly preferred α(2,6)-linked Neu5Ac when presented on Sialyl-Lac (Supplementary Fig. 2c,d, respectively). A similar binding preference was observed using sialylated trisaccharides containing a lactosamine core (Sialyl-LacNAc, 3′SLN and 6′SLN) (Supplementary Fig. 3).

It is noteworthy that the exact Neu5Ac linkage specificity for H1N1pdm remains unknown, with conflicting literature reports. Whilst a similar binding specificity for both α(2,3)- and α(2,6)-linked Neu5Ac has been previously reported^26,27^ it has also been demonstrated that H1N1pdm has a preference for α(2,6)-linkages^28^.

### Sialylated-Galβ(1,3)GalNAc *O*-glycoforms show different binding properties towards H1, H3, and H5 HA subtypes of influenza A virus

**Figure 3:**
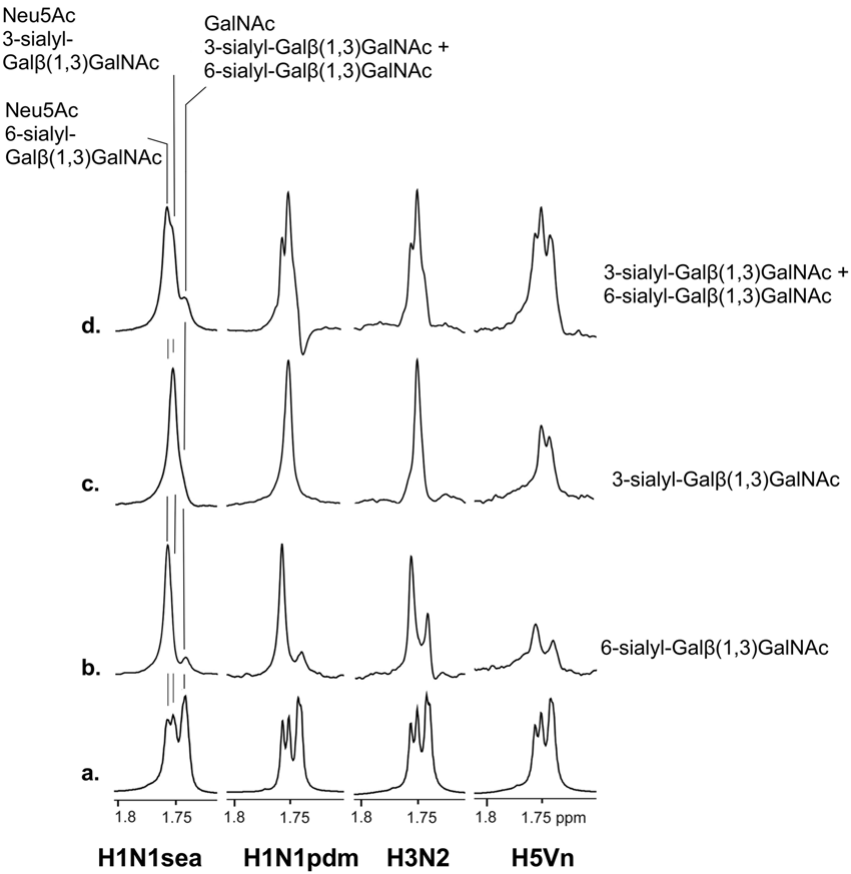
NMR spectra of influenza A viruses binding to mono-sialylated Galβ(1,3)GalNAc *O*-glycoforms: ^1^H NMR spectrum of an equimolar mixture of 3- and 6-Galβ(1,3)GalNAc (**a**) and STD NMR spectra of 6-sialyl Galβ(1,3)GalNAc (**b**), 3-sialyl Galβ(1,3)GalNAc (**c**) and an equimolar mixture of 3- and 6-sialyl Galβ(1,3)GalNAc (**d**), in complex with H1N1sea, H1N1pdm, H3N2 and H5Vn-VLP. The methyl protons of the acetamido groups of Neu5Ac and GalNAc are shown. For proposed binding epitope maps for sialyl-Galβ(1,3)GalNAc fragments see Supplementary Fig. 7.

**Figure 4:**
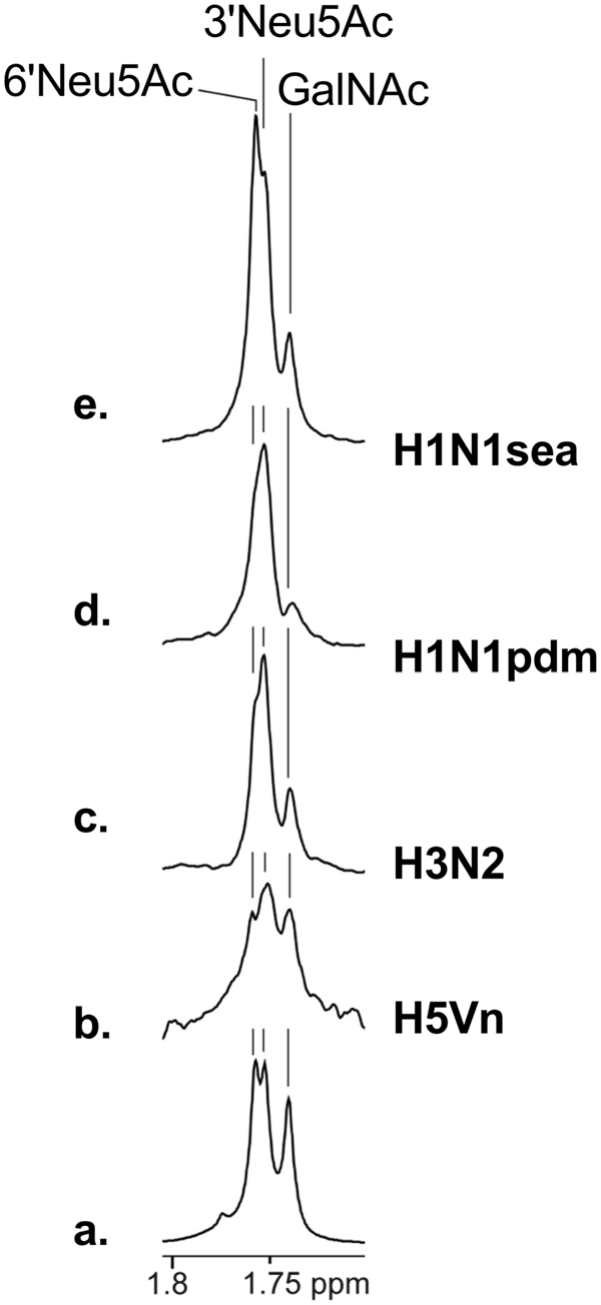
NMR spectra of influenza A viruses binding to di-sialylated Galβ(1,3)GalNAc: ^1^H NMR (**a**) and STD NMR spectra of 3,6-disialyl-Galβ(1,3)GalNAc in complex with H5Vn-VLP (**b**), H3N2 (**c**), H1N1pdm (**d**), and H1N1sea (**e**) are presented. The methyl protons of the acetamido groups of Neu5Ac and GalNAc are shown. For proposed binding epitope maps for sialyl-Galβ(1,3)GalNAc see Supplementary Fig. 7.

We then investigated the binding epitopes of 3-sialyl-Galβ(1,3)GalNAc, 6-sialyl-Galβ(1,3)GalNAc, and 3,6-disialyl-Galβ(1,3)GalNAc when bound to intact H1N1sea, H1N1pdm and H3N2 IAV particles and HA-VLPs derived from the avian H5N1 strain A/Vietnam/1203/2004(H5N1) (H5Vn-VLP). Figure 3 shows the STD NMR spectra of 3-sialyl-Galβ(1,3)GalNAc (Figs. 3c) and 6-sialyl-Galβ(1,3)GalNAc (Fig. 3b) in complex with viruses and VLPs revealing that the HAs from all investigated strains have affinity to all sialylated *O*-glycans used in this study. However, significant strain-specific differences in the observed glycointeractome could be detected. The entire spectra of 3-sialyl-Galβ(1,3)GalNAc and 6-sialyl-Galβ(1,3)GalNAc in complex with influenza virus strains are shown in Supplementary Fig. 4 and Supplementary Fig. 5, respectively. An STD NMR experiment of an equimolar mixture of 3-sialyl-Galβ(1,3)GalNAc and 6-sialyl-Galβ(1,3)GalNAc is shown in Fig. 3d. Remarkably the STD NMR signals for 3-sialyl-Galβ(1,3)GalNAc were stronger in the presence of H1N1pdm than the signals of 6-sialyl-Galβ(1,3)GalNAc suggesting that H1N1pdm prefers avian-like α(2,3)-linked Neu5Ac when present on *O*-glycans. The preference for α(2,3)-*O*-glycan receptors is in stark contrast to the observed and well described preference for human-like α(2,6)-linked receptors when presented as sialylated lactose or lactosamine^26,29,30^ as shown in Supplementary Fig. 2c and 3c. We could detect a preference for 3-sialyl-Galβ(1,3)GalNAc also for H3N2 whereas similar affinities for both α(2,3)- and α(2,6)-linked sialylated Lac were observed (Supplementary Fig. 2b). H1N1sea however interacted predominately with 6-sialyl-Galβ(1,3)GalNAc, similar to 6-sialylated fragments with a lactose core (Supplementary Fig. 2d). The STD NMR spectrum of H5Vn-VLPs showed a clear preference for 3-sialyl-Galβ(1,3)GalNAc, which is in complete agreement with a preference for 3-sialyl-lactose as published previously^23^. Most interesting is the observation that a significant binding contribution of the acetamido group of the GalNAc residue was also observed (Fig. 3, H5Vn). This result suggests significant differences in receptor recognition for H5Vn-VLPs, as the NMR spectra for human virus strains H1N1pdm, H1N1sea and H3N2 showed only little involvement of the acetamido methyl protons of the 6-sialyl-Galβ(1,3)GalNAc GalNAc residue (Fig. 3b) and weak, if any, STD NMR signal intensity for the GalNAc residue of 3-sialyl-Galβ(1,3)GalNAc (Fig. 3c). It is likely that this significantly distinct binding epitope of sialylated *O*-glycans for the avian subtype has a direct consequence for virus cell attachment and infection. The STD NMR experiments of the disialylated 3,6-disialyl-Galβ(1,3)GalNAc tetrasaccharide in complex with influenza viruses revealed a similar binding pattern to that observed for the equimolar mixture of 3- and 6-sialyl-Galβ(1,3)GalNAc (Fig. 4 and Supplementary Fig. 6). All HA subtypes show strong interaction with the acetamido methyl protons from the Neu5Ac units whereas only H5Vn-VLP showed similar interaction with the acetamido group of the GalNAc residue (Fig. 4b). This result also emphasizes the importance of the GalNAc residue in Sialyl-Galβ(1,3)GalNAc receptor recognition for the avian H5 subtype. The preference of H3N2 and H1N1pdm to bind α(2,3)-linked Neu5Ac was also detected for the 3,6-disialyl-Galβ(1,3)GalNAc (Fig. 4c,d, respectively). The determined epitope map (*glycointeractome*) for all sialylated sialylated core 1 oligosaccharides with the different influenza virus strains is shown in Supplementary Fig. 7. An important additional STD NMR experiment was acquired using the 3,6-disialyl-Galβ(1,3)GalNAc with biological relevant α-linked serine glycoside^17^. The STD NMR experiments using the H1N1pdm influenza virus strain (Supplementary Fig. 8) clearly shows that the glycointeractome of the disialylated *O*-glycan α-linked to serine is similar, if not identical, to the one described for the same glycan terminated by a β-linkage to the methyl aglycon. This result clearly supports our initial findings that the influenza virus binding epitope is determined by the sialylated core 1 fragment with the essential Galβ(1,3)GalNAc-linkage and not the configuration of the GalNAc residue. Two control STD NMR experiments were acquired to confirm the authenticity of the STD signals by using (*i*) a virus–glycan sample spiked with a non-binding sugar (sucrose) and (*ii*) heat-inactivated virus in complex with binding glycans (Supplementary Fig. 9).

### Influenza virus neutralization assays reveal a significant strain variation between H1 and H3 subtypes with regard to *O*-glycan binding

**Figure 5:**
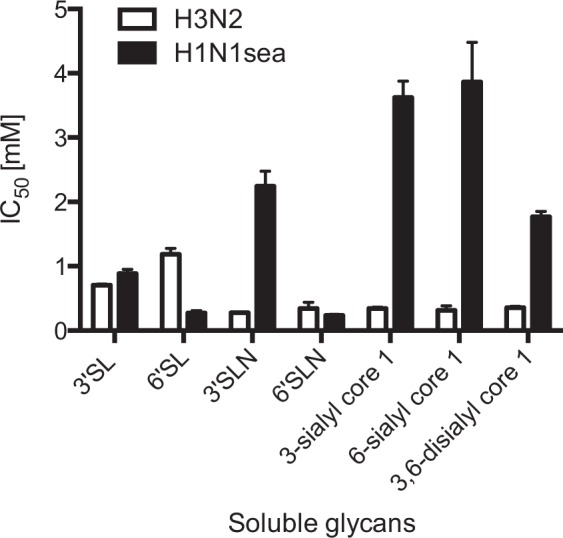
Comparison of IC_50_ values between H1N1sea and H3N2. The IC_50_ value is considered as the concentration of compound that reduced the focus forming ability by 50% compared to a non-treated infected cell monolayer. Two experiments with duplicates were performed. The error bars represent standard error of the mean.

To investigate the role of Sialyl-Galβ(1,3)GalNAc fragments during IAV cell attachment and infection, virus neutralization assays were performed in the presence of soluble glycans at various concentrations. The glycans act as decoy receptors by occupying the receptor binding sites of the HA protein, affecting the binding ability of the virus to the cell receptors depending on the affinity of the soluble glycans. Using a modified focus forming assay protocol, we were able to determine significant differences in IC_50_ values for 3-sialyl-Galβ(1,3)GalNAc, 6-sialyl-Galβ(1,3)GalNAc and 3,6-disialyl-Galβ(1,3)GalNAc between the two influenza strains H1N1sea and H3N2 (Fig. 5 and Table 1)^31^. All investigated *O*-glycoforms are strong competitors towards cell-receptors for H3N2, giving similar IC_50_ values to 3′SLN and 6′SLN (IC_50_ values of 0.28 and 0.35 mM, respectively). In contrast, infection with H1N1sea is less affected in the presence of the 3-sialyl-Galβ(1,3)GalNAc and 6-sialyl-Galβ(1,3)GalNAc (IC_50_ > 3.5 mM). Interestingly, 3,6-disialyl-Galβ(1,3)GalNAc is a stronger competitor than the monosialylated Galβ(1,3)GalNAc fragments with an IC_50_ value of 1.77 mM. Our results indicate a significant difference between the H1 and H3 subtypes with regard to *O*-glycan epitope binding. Furthermore, H1N1sea showed a distinct preference for α(2,6)-linked Neu5Ac in the Sialyl-LacNAc. 6′SLN (IC_50_ 0.24 mM) reduced cell infection significantly more than 3′SLN (IC_50_ 2.24 mM). In comparison, the inhibition level of H3N2 was similar for both α(2,3)- and α(2,6)-linked Neu5Ac in Sialyl-LacNAc, with IC_50_ values of 0.28 mM (3′SLN) and 0.35 mM (6′SLN), respectively. These results are in excellent agreement with our STD NMR results described above. Two negative controls [Galβ(1,3)GalNAc (=core 1) and sucrose] did not have any effect on both IAV strains with IC_50_ values of >20 mM each.

**Table 1:** Inhibition IC_50_ values [mM] of Sialyl-Lac/LacNAc in comparison to Sialyl-Galβ(1,3)GalNAc *O*-glycoforms towards H1N1sea and H3N2 influenza virus strains using a neutralization assay.

		IC_50_ [mM] H1N1sea	IC_50_ [mM] H3N2
Sialyl-Lac/LacNAc	**3′SL**	0.89 (±0.07)	0.71 (±0.02)
	**6′SL**	0.27 (±0.04)	1.19 (±0.09)
	**3′SLN**	2.24 (±0.23)	0.28 (±0)
	**6′SLN**	0.24 (±0.01)	0.35 (±0.1)
Sialyl-Galβ(1,3) GalNAc	**3-sialyl-Galβ(1,3)GalNAc (3-sialyl core 1)**	3.62 (±0.25)	0.35 (±0.02)
	**6-sialyl-Galβ(1,3)GalNAc (6-sialyl core 1)**	3.87 (±0.62)	0.31 (±0.07)
	**3,6-disialyl-Galβ(1,3)GalNAc (3,6-disialyl core 1)**	1.77 (±0.08)	0.36 (±0.02)
Controls	**Galβ(1,3)GalNAc**	≥20	≥20
	**Sucrose**	≥20	≥20

The errors are represented as standard error of the mean.

### Inhibition of *O*-glycan biosynthesis in Lec1 cells affects 3045/H5N1 influenza virus infection

**Figure 6:**
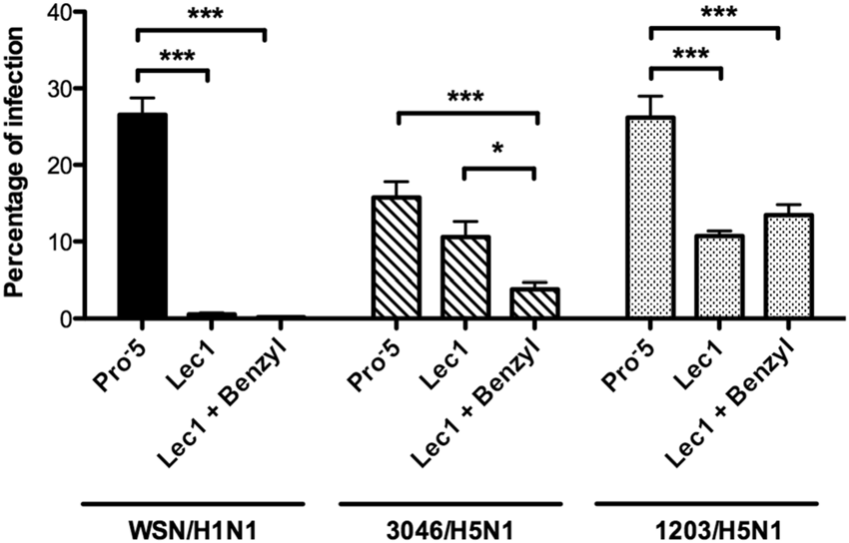
Level of infection (%) of Pro^−^5 cells and Lec1 cells with WSN33/H1N1 and H5N1 viruses before and after treatment with GalNAcα-*O*-Bn (Benzyl). ***(*p* < 0.0001) and *(*p* < 0.05) shows there is a statistically different decrease in infection between the two cell types and the use of GalNAcα-*O*-Bn. The percentage of infection was calculated using Aperio image analysis software. The same colour/pattern of the bar represents influenza viruses of the same subtype.

Lec1 CHO cells, which lack the *N*-acetylglucosaminyl-transferase I (GlcNAcT-I) activity necessary for the formation of complex or hybrid *N*-glycans^32^, were used to further elucidate the role of *O*-linked glycans in avian IAV infection. Lec1 cells were also treated with GalNAc-α*O*-Bn to inhibit their *O*-glycan glycoprotein but not glycolipid biosynthesis. By comparing the influenza infection rate in the parental CHO (Pro^−^5) cells and the Lec1 cells, the significance of *O*-linked glycans could be ascertained. Fig. 6 and Table 2 show that there was minimal infection by WSN33/H1N1 in the Lec1 cells (0.57% ± 0.34%), but significant infection by 3045/H5N1 (10.57% ± 3.54%) and 1203/H5N1 (10.73% ± 1.58%), similar to previous findings^33^. However, a reduced infection by 3045/H5N1 (3.83% ± 2.2%, *p* < 0.0001) in Lec1 cells treated with GalNAcα-*O*-Bn in comparison to control Lec1 cells was observed. The reduced infection levels indicated the biologically relevant role of α(2,3)-sialylated *O*-glycans in some H5N1 virus infections, although it was not observed in 1203/H5N1 infection suggesting that the Neu5Acα(2,3)Galβ(1,3)GalNAc epitope present in some glycolipids such as G_D1a_ and G_M1b_ may be involved in binding of this H5N1 strain.

**Table 2:** Level of infection (%) of Pro^−^5 cells and Lec1 cells with WSN33/H1N1, 3045/H5N1 and 1203/H5N1 viruses before and after treatment with GalNAcα-*O*-Bn (Benzyl).

[%]	WSN33/H1N1			3045/H5N1			1203/H5N1		
	Pro^−^5	Lec1	Lec1 + Benzyl	Pro^−^5	Lec1	Lec1 + Benzyl	Pro^−^5	Lec1	Lec1 + Benzyl
Mean	26.56	0.57	0.18	15.77	10.57	3.83	26.18	10.73	13.44
Std. Deviation	3.79	0.34	0.06	3.53	3.54	2.20	6.84	1.58	3.34
Std. Error of Mean	2.18	0.19	0.03	2.04	2.05	0.89	2.79	0.65	1.36

### Structural analysis and Molecular Dynamics (MD) simulations of sialylated glycans in complex with HA from pandemic H1N1 and avian H5N1

**Figure 7:**
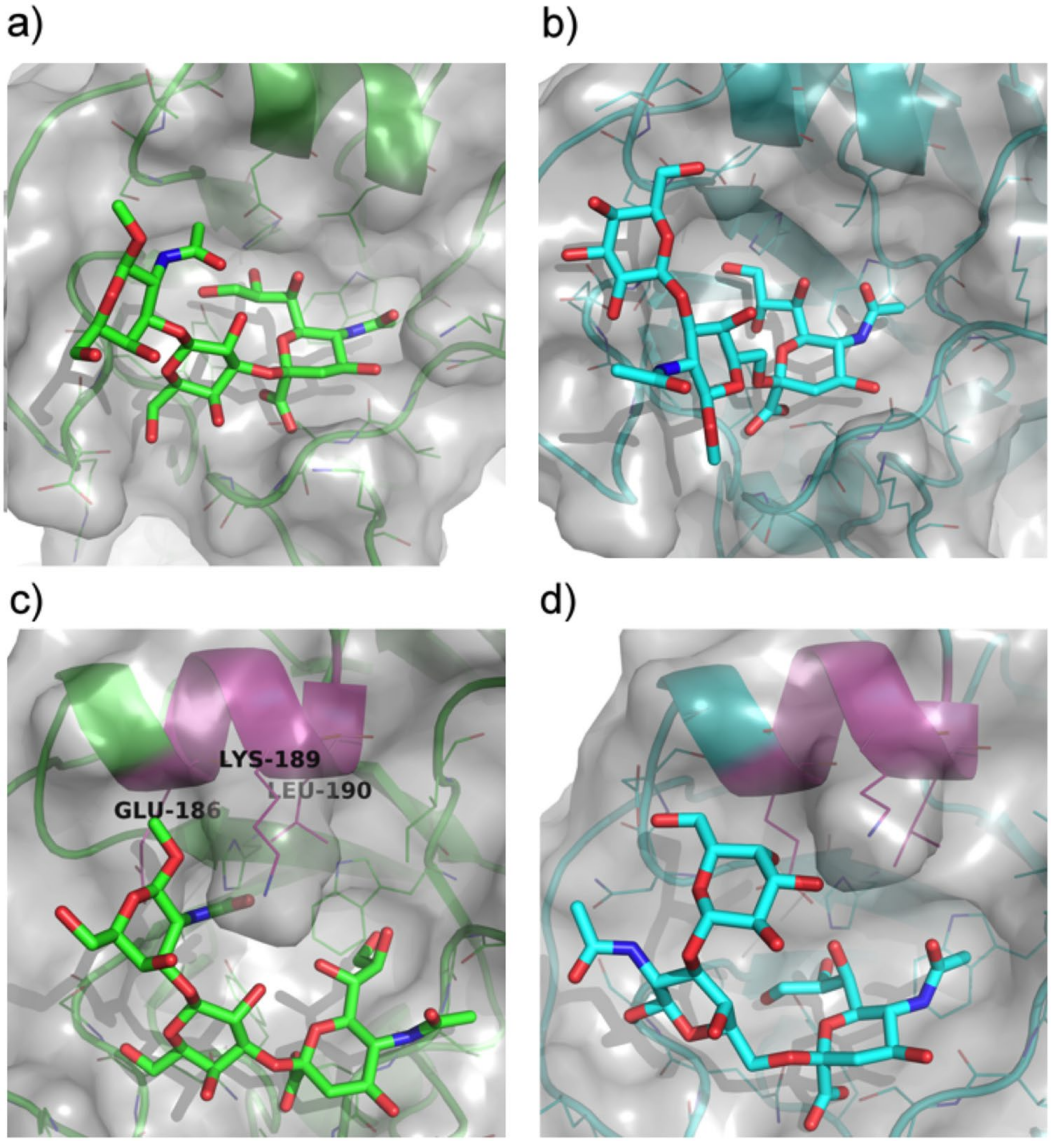
Molecular Dynamics (MD)-derived structures of 3- and 6-sialyl-Galβ(1,3)GalNAc (**a,b**, respectively) bound to the HA from A/California/04/2009(H1N1) (H1N1pdm) and MD-derived structures of 3- and 6-sialyl-Galβ(1,3)GalNAc (**c** and **d**, respectively) bound to HA from avian A/Vietnam/1194/2004(H5N1).

To explain the receptor specificity-shift of H1N1pdm-HA from α(2,6)- to α(2,3)-linked Neu5Ac, its X-ray crystal structure in complex with 6′SLN (PDB: 3UBN)^29^ was used as a starting structure to obtain a bound structure of 3-sialyl-Galβ(1,3)GalNAc via molecular docking. The docked structure with the best superimposition between the Neu5Ac moiety of 3-sialyl-Galβ(1,3)GalNAc and 6′SLN was subjected to further optimization by a 20 ns MD simulation. The final snapshot of this MD simulation reveals a potential bound conformation of 3-sialyl-Galβ(1,3)GalNAc (Fig. 7a). The Neu5Ac and Gal residues of 3-sialyl-Galβ(1–3)GalNAc maintain key interactions with the protein when compared to 6′SLN (Supplementary Fig. 10). In particular, the methyl protons of the acetamido group of Neu5Ac are engaged in strong hydrophobic contacts with the indole ring of Trp-104 (Supplementary Fig. 11) similar to the carbohydrate-aromatic interactions previously reviewed^34^. In contrast, the GalNAc residue was involved in some interaction with the protein. The methyl protons of the acetamido group of the GalNAc residue established no direct contact with the protein, which is in excellent agreement with our STD NMR results. This result further suggests that, despite the difference in the Sia glycosidic linkages, the HA protein from the H1N1pdm can accommodate a sialylated Galβ(1,3)GalNAc core without any steric hindrance. A combined docking and MD simulation of the H1N1pdm HA in complex with 6-sialyl-Galβ(1,3)GalNAc was also performed illustrating a ‘*loose’* bound conformation of 6-sialyl-Galβ(1,3)GalNAc with the HA protein (Fig. 7b). The major 6-sialyl-Galβ(1,3)GalNAc–HA interactions can be attributed to the Neu5Ac residue, whereas the remainder of the glycan core does not make any significant contact with the protein. This *loose* binding conformation of 6-sialyl-Galβ(1,3)GalNAc is likely the reason that H1N1pdm has binding preference for 3-sialyl-Galβ(1,3)GalNAc which is in excellent agreement with the STD NMR experiments demonstrating that 3-sialyl-Galβ(1,3)GalNAc is clearly the preferred bound sialylated Galβ(1,3)GalNAc fragment.

**Figure 8:**
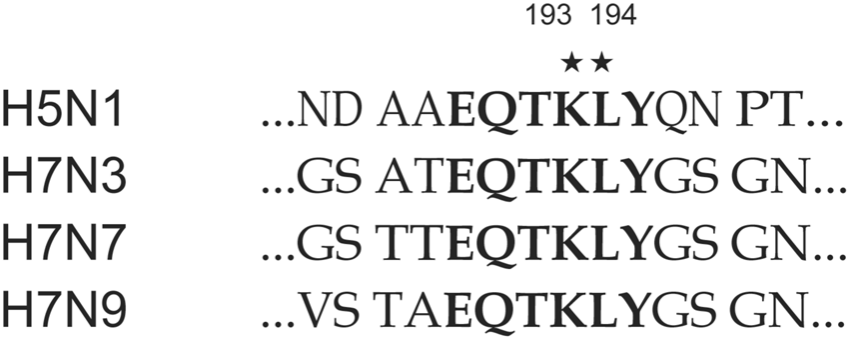
Identification of an EQTKLY motif in Highly Pathogenic Avian Influenza (HPAI) strains. Multiple protein sequence alignment of the region containing the crucial GalNAc-binding amino acid residues Lys-193 and Leu-194 (H3 numbering) (★) of several HPAI strains. The two residues can be found within a sequence motif consisting of six amino acids EQTKLY (shown in bold) being highly conserved in all HPAI strains used in this alignment (see Supplementary Fig. 13 for the entire sequence alignment). This sequence motif is not conserved in the human IAV strains H1N1pdm, H1N1sea and H3N2 (alignment not shown).

We have also docked 3-sialyl-Galβ(1,3)GalNAc into the binding site of the HA of A/Vietnam/1194/2004(H5N1) (PDB: 3ZNK)^35^. The 3-sialyl-Galβ(1,3)GalNAc structure with the best superimposition of the Neu5Ac residue with that of the crystal structure bound ligand 6-*O*-sulfated 3′sialyllactosamine [Neu5Acα(2,3)Galβ(1,4)(6-HSO_3_)GlcNAc], was chosen for a subsequent 20 ns MD simulation, which illustrated that GalNAc of 3-sialyl-Galβ(1,3)GalNAc is significantly engaged in interaction with the HA protein (Fig. 7c). In particular, the acetamido group of the GalNAc residue makes a strong polar interaction with Lys-189 as well as a hydrophobic interaction with Leu-190 (Lys-193 and Leu-194 according to H3 numbering). This finding is also in excellent agreement with the STD NMR results showing that the methyl protons of the acetamido group of the GalNAc residue are important for 3-sialyl-Galβ(1,3)GalNAc binding. Likewise, an MD structure for the bound conformation of 6-sialyl-Galβ(1,3)GalNAc was obtained (Fig. 7d). Although its Neu5Ac moiety maintained interactions with the HA protein similar to α(2,3)-linked *O*- and *N*-glycans, both the Gal and GalNAc portions of 6-sialyl-Galβ(1,3)GalNAc were more solvent-exposed and established less contacts with the protein than those of 3-sialyl-Galβ(1,3)GalNAc. These observations are consistent with the overall weak absolute STD enhancements of 6-sialyl-Galβ(1,3)GalNAc (Supplementary Fig. 5d). Our analysis suggests that sialylated Galβ(1,3)GalNAc fragments are potential receptors for avian H5N1 influenza virus due to additional strong interaction with the acetamido group of the GalNAc residue. Our discovery that sialylated Galβ(1,3)GalNAc fragments are potential receptors for H5N1 has promoted us to compare the amino acid sequence of the HA of several HPAI strains (Fig. 8). We identified a sequence motif EQTKLY consisting of six amino acids including the crucial GalNAc-binding residues Lys-193 and Leu-194 that is highly conserved in H5N1, H7N3, H7N7 and H7N9. We postulate that the EQTKLY-motif is essential in coordinating the GalNAc residue in *O*-glycans for successful binding to HPAI strains. Additionally, we have performed docking studies of the 3,6-disialyl core 1 α-linked to serine that shows that the α-serine residue is completely solvent exposed, supporting the NMR experiments (Supplementary Fig. 12).

Our results show that sialylated Galβ(1,3)GalNAc glycoforms are not only recognized and bound by several IAV strains, but also affect viral infection cycles. Sialylated core 1 *O*-glycans have been found previously in the respiratory tract tissues of ferrets and humans^10,11^. In the current study we show expression of ST6GalNAc1, a key enzyme in α(2,6)sialylated *O*-glycan biosynthesis, in A549 cells and type I pneumocytes, as well as in human nasopharyngeal and lower respiratory tract tissues including bronchus, bronchioles and alveoli.

The observation that the acetamido group of the GalNAc residue of sialyl-core 1 *O*- glycans is bound with similar affinity to the acetamido group of the Neu5Ac moiety by avian H5, suggests that sialylated Galβ(1,3)GalNAc fragments might be essential receptors for this subtype. This outcome correlates very well with binding data from glycan microarray assays^36,37^ and the results reported by Gaunitz and co-workers showing strong binding of H5Vn protein to 3-sialyl-Galβ(1,3)GalNAc by SPR technology^38^. Our structural investigation presented here shows that the GalNAc moiety of the *O*-glycan core 1 structure is coordinated by Lys-193 and Leu-194. Both amino acid residues lie within the EQTKLY-motif that is highly conserved in HPAI strains but not in human virus HA proteins of the H1 and H3 subtypes. This structural observation correlates well with our NMR finding that proteins of human strains that lack the EQTKLY-motif show only weak, if any, interaction with the acetamido group of the GalNAc moiety and therefore reduced binding. The question of why avian H5N1 influenza viruses are less effective in human transmission may also be explained by the high prevalence of sialylated motifs in mucins. It is likely that the sialylated *O*-glycan motifs present in mucins restrict access to apical receptors expressed on the respiratory epithelium, suggesting a dual role for exposed mucin *O*-glycans in masking specific cell-surface receptors and acting as a decoy. It has been reported that mucin *O*-glycans prevent bacterial adhesion by limiting access to the epithelial cell surface^39^.

The second significant finding of our study is that the presence of the Galβ(1,3)GalNAc disaccharide in sialylated *O*-glycan motifs causes the human H1N1pdm virus to switch its preference from α(2,6)-linked Neu5Ac to α(2,3)-linked Neu5Ac (‘*avian receptor*’). The different impact of the neighbouring glycan residues on recognition of sialic acid as a receptor for influenza virus has been observed before^40^.

The virus neutralization assays also revealed strain-specific differences in the biological importance of sialylated core 1 *O*-glycan oligosaccharides to influenza H1N1sea and H3N2 subtypes. All investigated *O*-glycan oligosaccharides showed strong inhibitory potency towards human H3N2 influenza virus. In contrast, H1N1sea infection of MDCK.2 cells was less adversely affected by sialylated core 1 structures; for example the soluble *O*-glycan 6-Sialyl-Galβ(1,3)GalNAc fragment was approximately 14-fold less effective than 6-SL/SLN in competing with glycan receptors on the host cell surface. However, sialylated *O*-glycoforms clearly bound to H1N1sea suggesting that they may act like attachment factors to support initiation of the infection cycle. We have demonstrated for the first time that particular avian H5 influenza strains can infect CHO Lec1 cells via *O*-glycan receptors. Chu and Whittaker postulated that influenza viruses could not infect CHO Lec1 cells due to the lack of complex and sialylated *N*-glycans^33^, however in that study influenza viruses A/WSN/33(H1N1), Udorn/307/72(H3N2) and influenza B/Yamagata/78 were used, but no avian influenza strains were tested.

In conclusion, our study sheds light on the involvement of the Sialyl-Galβ(1,3)GalNAc *O*-glycoforms during IAV infection, suggesting a similar important role as Sialyl-Lac/LacNAc motifs for certain influenza strains. Furthermore, it provides the first evidence of a non-sialic acid residue, namely *O*-glycan-specific GalNAc, playing a crucial role in HPAI pathogenesis. This outcome opens a new direction for structure-assisted drug design and may facilitate the development of potent anti-influenza drugs especially towards HPAI strains that threaten the human population.

## Methods

### Immunocytochemistry and image analysis

Coverslips were washed with TBS and 0.1% Tween 20 (Sigma P-1379) for 30 min at room temperature (RT) then blocked with 10% normal rabbit serum for 10 min. They were then incubated with 1/25 HB65 for 30 min at RT followed by 1/200 biotinylated rabbit anti-mouse (Dako Cytomation E-0354) for 30 min at RT and incubated with Elite-ABC kit (Vectorlab PK-6100) diluted 1/50 for 30 min at RT. Colour was developed with the Vector Red substrate kit (Vector Labs SK-5100) and counterstaining with Mayer’s hematoxylin. Slides were scanned using Aperio Scanscope CS-S microscopic slide scanning system and proprietary image analysis counting the number of positive cells as a percentage of the total cell population with on average 6,000 to 12,000 cells counted/region and three regions per coverslip assessed (mean ± standard deviation). For staining with sialyltransferase antibodies coverslips were transferred to PBS then treated with 0.1% Tween 20 in TBS for 30 min RT then blocked with 2.5% normal horse serum (NH) for 5 min at RT. Primary antibody was applied (ST6GAL1 (R&D System AF5924), ST6GalNAc1 (Novus NBP1–87043), and ST6GalNAc2 (Novus NBP2–13392)) followed by biotinylated horse anti-goat IgG, then streptavidin, alkaline phosphatase conjugated 1/50, and development with VECTOR Red alkaline phosphatase (AP) substrate kit with levamisole solution added followed by counterstaining with Mayer’s hematoxylin. Sections were stained using the same primary antibodies following antigen retrieval using microwave sections in 10 mM citrate buffer pH 6.0 at 95 °C for 15 min.

### H5Vn-VLP preparation

HEK293T cells were transfected with pCMVdR8.91 (coding for HIV- Gag/Pol gene) and pcDNA-synH5 from a H5N1 known clinical isolate grown in the presence of soluble *Vibrio cholerae* sialidase (4 mU/mL; Roche). Supernatant was harvested 24 h post-transfection, filtered and concentrated on sucrose cushion (20% w/v of sucrose (Sigma, S-7903) filter sterilized on 0.45 um). After ultra-centrifugation (at 28,000 × g for 2.5 h on Optima L80 XP from Beckman Coulter equipped with rotor SW 32 TI), the pellet was re-suspended into 1/100th of the initial volume of the complete Dulbecco’s Modified Eagle’s Medium (DMEM/HIGH, Invitrogen #10569), 5% foetal bovine serum (FBS, Invitrogen #10500) and 1% Penicillin/ streptomycin (Invitrogen #15140). As the haemagglutinin construct contains a flag-tag on its C-terminal end, the content of HA in these VLP’s could be established by western-blot using anti-flag M2 peroxidase conjugate monoclonal antibodies (Sigma, A-8592) with a calibrating serial dilution of BAP-Flag protein (Sigma, P-7582).

### Virus neutralization assay

The neutralization assay was performed using a modified focus forming assay protocol^31^. 3 × 10^4^ MDCK.2 cells were seeded into 96-wells and incubated at 37 °C for 24 hrs. The following steps were performed at 4 °C to inhibit the neuraminidase activity, as the addition of oseltamivir carboxylate (OC) to block neuraminidase was not suitable in these assays because an efficient spread of virus progeny to the neighbouring cells is essential to obtain countable foci for the analysis. Confluent MDCK monolayers were washed with cold DMEM without additives. 100 mM stock solution of all glycans were prepared in ultrapure water and sterilized by irradiating under UV light on ice for 20 min. 3′SL, 6′SL, 3′SLN and 6′SLN were used as positive controls and sucrose and core 1 *O*-glycan motif Galβ(1,3)GalNAc as negative controls. Concentration of 0, 0.2, 0.6, 1, 2, 5 and 10 mM of each glycan were initially tested followed by a more refined concentration range of 0, 0.1, 0.2, 0.3, 0.4, 0.5, 0.6, 1 mM if required. Virus (150–200 FFU/well, to be determined for each strain) and glycan were mixed in infection media (DMEM + 2 mM L-glutamine + 0.1% BSA) and incubated for 30 min on ice before addition to cells monolayers for 3 hrs at 4 °C with regular rocking. As a positive control, cell monolayers were incubated with the same virus titer minus glycans. After removal of unbound virus by carefully pipetting off the supernatant and washing of the cell monolayer with cold DMEM without additives, the cells were covered with the Avicell-overlay (1.25% Avicell RC-591 + DMEM + 2 mM L-glutamine + 0.1% BSA + 1 μg/ml TPCK-trypsin) and incubated at 35 °C for 20 hrs. After propagation of virus, the Avicell-overlay was carefully removed and the monolayer was fixed with 4% paraformaldehyde, MetOH free (Pierce # 28908) at RT for 15 min. The wells were washed 3 × 5 min with PBS and permeabilised with 0.5% Triton X-100 for 10 min was used for permeabilisation followed by another washing step. H_2_O_2_ treatment with 0.3% H_2_O_2_ in PBS for 30 min in dark was performed to inactivate endogenous peroxidase and reduce background. Primary antibody anti-Influenza A NP mab (final 1 μg/ml; AbD Serotec Influenza A NP mAb, MCA400) in PBS + 1% BSA + 0.05% Tween 20 was then incubated for 1 hr before washing with PBS + 0.05% Tween 20. Secondary antibody goat-anti-mouse IgG-HRP (final 1 μg/ml; BioRad 172–1011) in PBS + 1% BSA + 0.05% Tween 20 was incubated for 1 hr and washed off 4 × 5 min with PBS + 0.05% Tween 20. IAV infected cells were visualised with TrueBlue reagent (KPL 70–00–64) according manufacturer’s instructions and blue foci containing five or more infected cells were manually counted under an Olympus CKX31 inverted microscope. Data analysis and calculation of virus neutralization IC_50_ values (non-linear regression (curve fit), dose-response inhibition, four parameter logistic) were carried out using GraphPad Prism 6 (GraphPad Software, Inc., La Jolla, CA). The IC_50_ value was considered as the concentration of glycan that reduced the focus forming ability by 50% compared to a non-treated infected cell monolayer. Two individual experiments with duplicates were performed. The data represents two biologically independent experiments in duplicate, and the error bars represent the standard error of the mean.

### Virus NMR experiments

For STD NMR experiments, purified viruses were UV-inactivated for 10 min and buffer exchanged to 20 mM phosphate buffer pH 7.2 and 70 mM NaCl in D_2_O using a 100 kDa Amicon (Millipore) centrifugal device. The glycans of interest were solved in D_2_O. Influenza virus preparations were incubated with 50 μM oseltamivir carboxylate for 10 min at RT to block the neuraminidase activity, followed by the addition of 3 mM of glycan. All STD NMR spectra were acquired in Shigemi tubes (Shigemi, USA) with a Bruker 600 MHz Advance spectrometer at 283 K using ^1^H/^13^C/^15^N cryoprobe equipped with z-gradients. Virus particles were saturated at −1.0 ppm (on-resonance) and 300 ppm (off-resonance) using a cascade of 60 selective Gaussian-shaped pulses of 50 ms duration. A 100 µs delay between each pulse was applied, resulting in a total saturation time of 3 s. A relaxation delay of 4 s was used. A total of 1512 scans per STD NMR experiment were acquired and a WATERGATE sequence was used to suppress the residual HDO signal. Spin-lock filter with 5 kHz strength and duration of 10 ms was applied to suppress protein background. On- and off-resonance spectra were stored and processed separately, and the final STD NMR spectra were obtained by subtracting the on- and off-resonance spectra. Control STD NMR experiments were performed with an identical setup but with sucrose as a non-binding control glycan as well with heat-inactivated virus (20 min at 70 °C). Before and after the STD NMR experiment, ^1^H NMR spectra were recorded as control to ensure the integrity of the samples.

### Carbohydrates and reagents

Deuterium oxide was purchased from Sigma Aldrich (Australia). Sialylα(2,3)Galβ(1,4)Glc (3′Sialyllactose, 3′SL), Sialylα(2,6)Galβ(1,4)Glc (6′Sialyllactose, 6′SL) were purchased from Elicityl (France). (3′Sialyllactosamine, 3′SLN), Sialylα(2,6)Galβ(1,4)GlcNAc (6′Sialyllactosamine, 6′SLN) were purchased from Elicityl (France). Benzyl-2-acetamido-2-deoxy-α-d-galactopyranoside (GalNAcα-O-Bn) was purchased from Carbosynth (Berkshire, UK).

### Synthesis of sialylated *O*-glycoforms

#### General methods

Reactions were monitored by thin layer chromatography (TLC) using aluminium plates coated with Silica Gel 60 F254 (Merck). Detection was typically effected under ultraviolet (uv) light where applicable, followed by treatment with H_2_SO_4_ in EtOH (5% v/v) and charring at ~200 °C. Purification by flash chromatography was achieved by elution through columns of silica gel 60 (0.040–0.063 mm). Waters Sep-Pak Vac C18 Cartridges were used to purify final compounds. ^1^H and ^13^C NMR spectra were recorded using a Bruker Avance 300 MHz or 600 MHz spectrometer. For ^1^H and ^13^C NMR spectra, chemical shifts are expressed as parts per million (ppm, δ) and are relative to the residual solvent peak [D_2_O 4.79 (s) for ^1^H]. Multiplicities are denoted as s (singlet), d (doublet), t (triplet), dd (doublet of doublets), m (multiplet), app (apparent). 2D NMR experiments were performed using ^1^H-^1^H correlation spectroscopy (COSY) and ^1^H-^13^C Heteronuclear Single Quantum Coherence (HSQC) to confirm ^1^H and ^13^C assignments. High-Resolution Mass Spectrometry (HRMS) was carried out on an Agilent 1290 HPLC/6530 QTOF with a Jet stream ESI source. All chemicals except otherwise stated, were purchased from Sigma-Aldrich with the highest purity available. *N-*Acetylneuraminic acid (5-acetamido-3,5-dideoxy-d-*glycero*-d-*galacto*-non-2-ulosonic acid) was obtained from Carbosynth Limited (UK). Enzymes were purchased from Chemily (USA).

### Molecular dynamics (MD) simulations

The initial structures for MD simulations were obtained by molecule docking of 3-sialyl and 6-sialyl core 1 *O*-glycans with AutoDock Vina^41^. The X-ray crystal structures of the HA protein from IAV strain H1N1/A/California/04/2009 (H1N1pdm) (PDB: 3UBN^29^,) and H5N1/A/Vietnam/1203/2004 (PDB: 3ZNK)^35^ were used as receptors for molecular docking. Amber ff14SB^42^ and Glycam06^43^ force fields were applied to the protein and glycans, respectively. MD simulations of 20 ns length were carried out using GROMACS^44^. The final snapshot of MD trajectories was used for analyses.

## Electronic supplementary material

Supplementary Information
