# Supplementary material for: Unravelling the Role of O-glycans in Influenza A Virus Infection
Source: Sci Rep. 2018 Nov 6;8:16382. doi: 10.1038/s41598-018-34175-3 (PMC6219607; doi:10.1038/s41598-018-34175-3)
Supplement: Supplementary file 1 — Supplementary Information [file 41598_2018_34175_MOESM1_ESM.pdf]

Unravelling the Role of *O*-glycans in Influenza A Virus Infection

Juliane Mayr[1#], Kam Lau[1#], Jimmy C. C. Lai[2], Ivan A. Gagarinov[1], Yun Shi[1], Sarah McAtamney[1], Renee W. Y. Chan[3], , John Nicholls[2], Mark von Itzstein[1]* and Thomas Haselhorst[1]*

Supplementary Information and Figures

Supplementary Figures

A

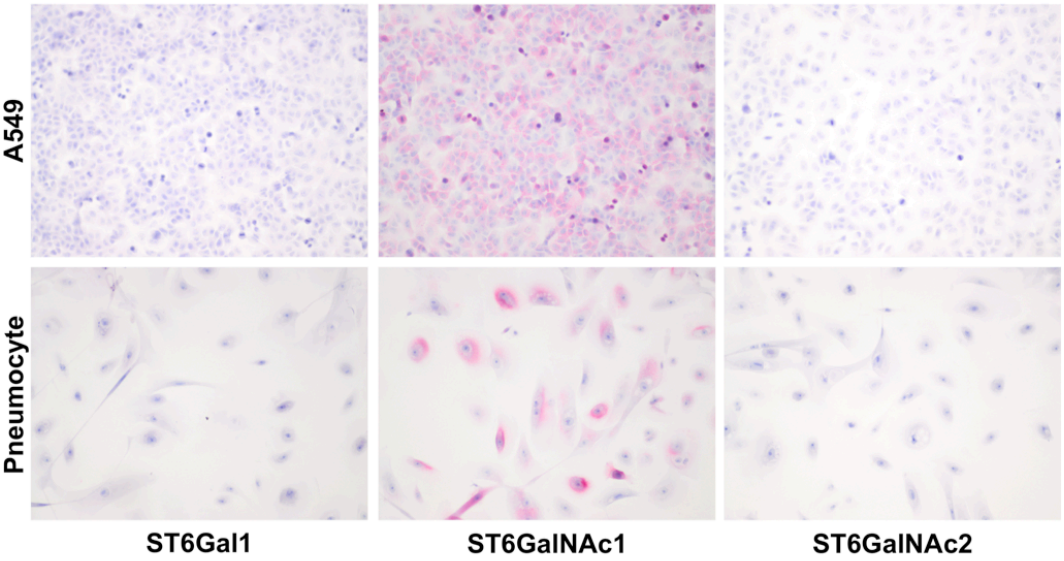

B

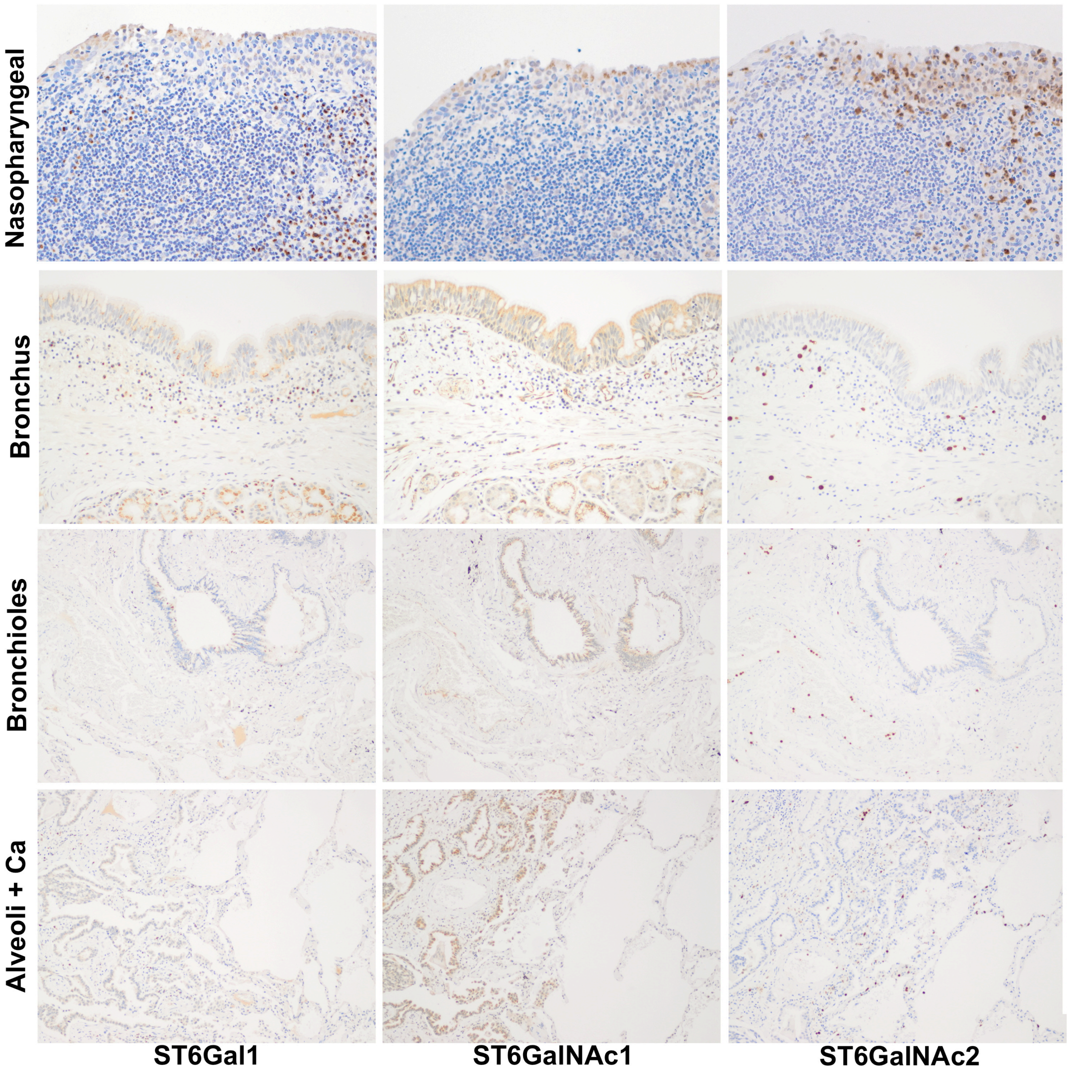

Supplementary Figure 1: (**A**) Immunocytochemistry of A549 and type 1 like pneumocytes. (**B**) immunohistochemistry of normal human nasopharyngeal, bronchus, bronchioles and alveolar epithelium for the expression of ST6Gal1, ST6GalNAc1 and ST6GalNAc2. Positive cells are red (**A**) or brown (**B**) in colour and counterstained with haematoxylin. Magnification of coverslips is 200x and of histological sections is 100x.

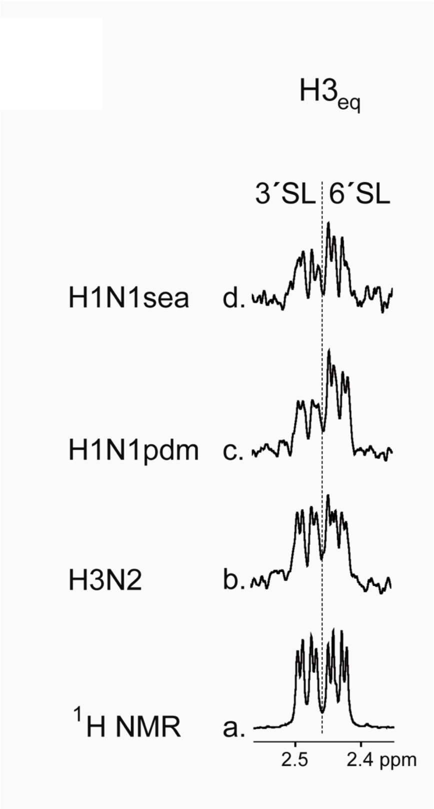

Supplementary Figure 2: NMR spectra of influenza A viruses binding to 3'- and 6'-sialyllactose (3'SL and 6'SL). ^{1}H NMR spectrum of an equimolar mixture of 3 mM 3'SL and 6'SL (**a**) and STD NMR spectra in the presence of H3N2 (**b**), H1N1pdm (**c**), and H1N1sea (**d**). Only signals of the H3eq of *N*-acetylneuraminic acid are shown.

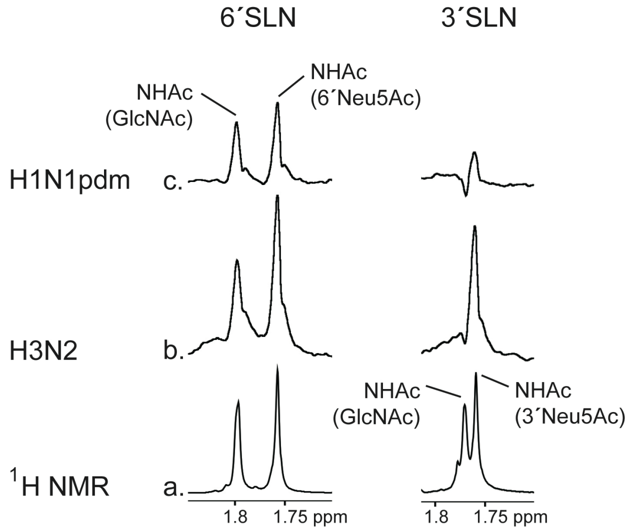

Supplementary Figure 3: Influenza A virus binding to 3'SLN and 6'SLN: ^{1}H NMR spectra (a) and STD NMR spectra of H3N2 (b) and H1N1pdm (c) influenza virus in the presence of 3 mM 6'SLN and 3'SLN. Strong STD NMR signals could be detected for the methyl protons of the two acetamido groups (Neu5Ac and GlcNAc) of 6'SLN when bound to H1N1pdm (lane c/left). In stark contrast, the STD NMR spectrum obtained with 3'SLN in complex with H1N1pdm revealed only weak interaction without engagement of the acetamido group of the GlcNAc moiety (lane c/right). This is in excellent agreement with the X-ray crystal structure showing that 6'SLN attains a very 'compact' conformation (pdb: 3UBN) and the *N*-acetylneuraminic acid (Neu5Ac), galactose (Gal) and *N*-acetylglucosamine (GlcNAc) make strong contact with the protein surface. H3N2 shows binding to both 3'SLN and 6'SLN with a comparable specificity as already shown for 3'SL and 6'SL (supplementary figure 2b).

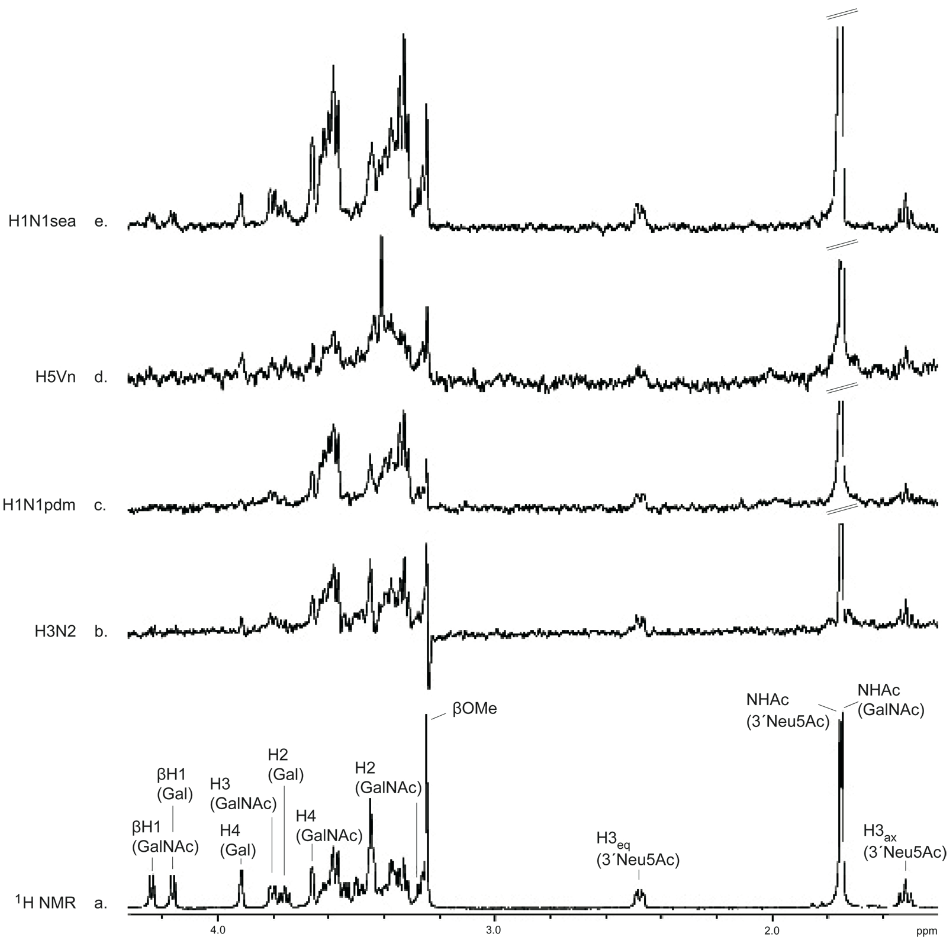

Supplementary Figure 4: Influenza A virus binding to 3-sialyl-Galβ1-3GalNAc. ^{1}H NMR spectra (a) and STD NMR spectra of H3N2 (b), H1N1pdm (c), H5Vn-VLP (d) and H1N1sea (e) influenza virus in the presence of 3 mM 3-sialyl-Galβ1-3GalNAc are shown. The assigned peaks of 3-sialyl-Galβ1-3GalNAc are labeled in the ^{1}H NMR spectrum (a).

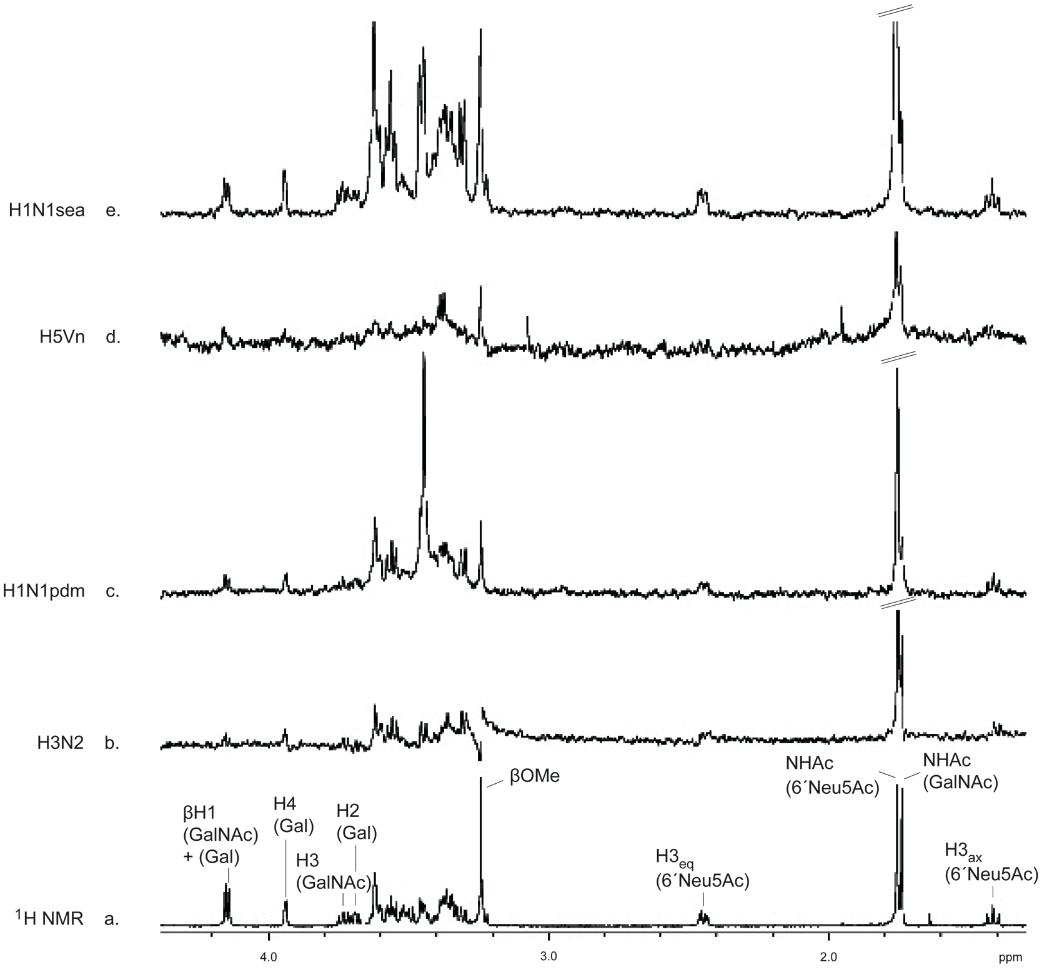

Supplementary Figure 5: Influenza A virus binding to 6-sialyl-Galβ1-3GalNAc. ^{1}H NMR spectra (a) and STD NMR spectra of H3N2 (b), H1N1pdm (c) H5Vn-VLP (d) and H1N1sea (e) influenza virus in the presence of 3 mM 6-sialyl-Galβ1-3GalNAc are shown. The assigned peaks of 6-sialyl-Galβ1-3GalNAc are labelled in the ^{1}H NMR spectrum (a).

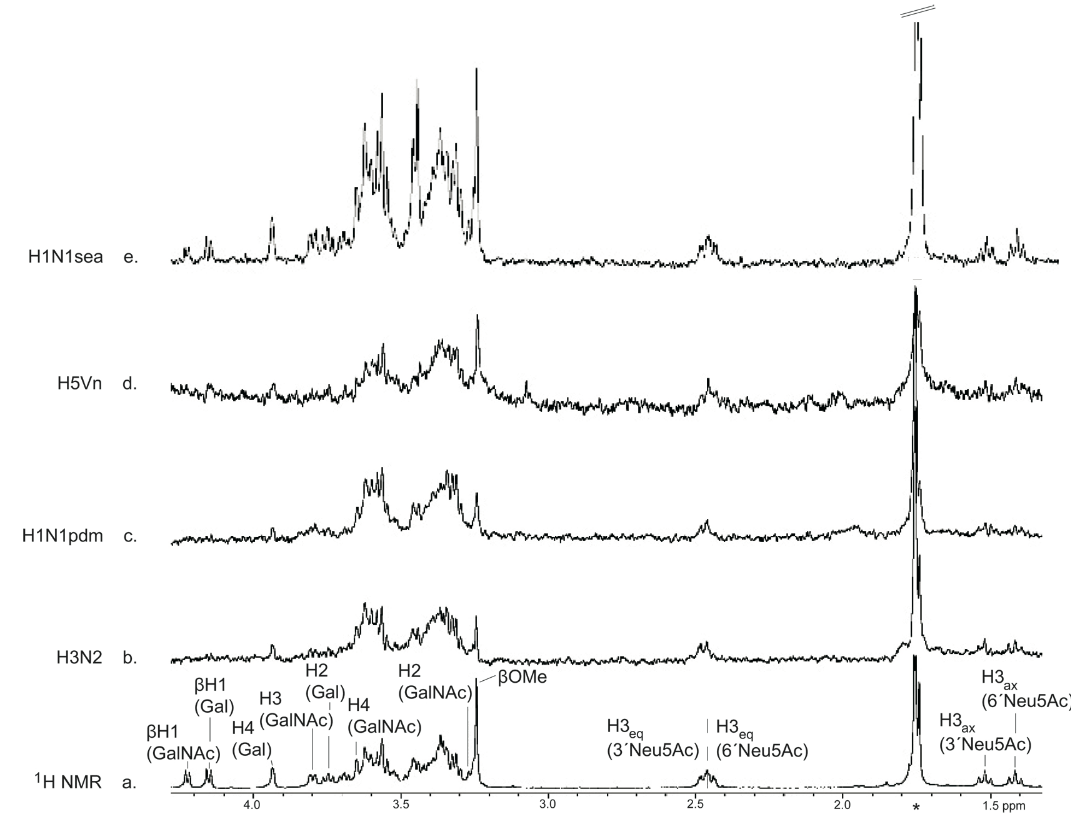

Supplementary Figure 6: Influenza A virus binding to 3,6-disialyl-Galβ1-3GalNAc. ^{1}H NMR spectra (a) and STD NMR spectra of H3N2 (b), H1N1pdm (c) H5Vn-VLP (d) and H1N1sea (e) influenza virus in the presence of ,6-disialyl-Galβ1-3GalNAc. The assigned peaks of 3,6-disialyl-Galβ1-3GalNAc are labelled in the ^{1}H NMR spectrum (a). (*) For peak assignment of the NHAc peaks please refer to figure 4 in the main publication.

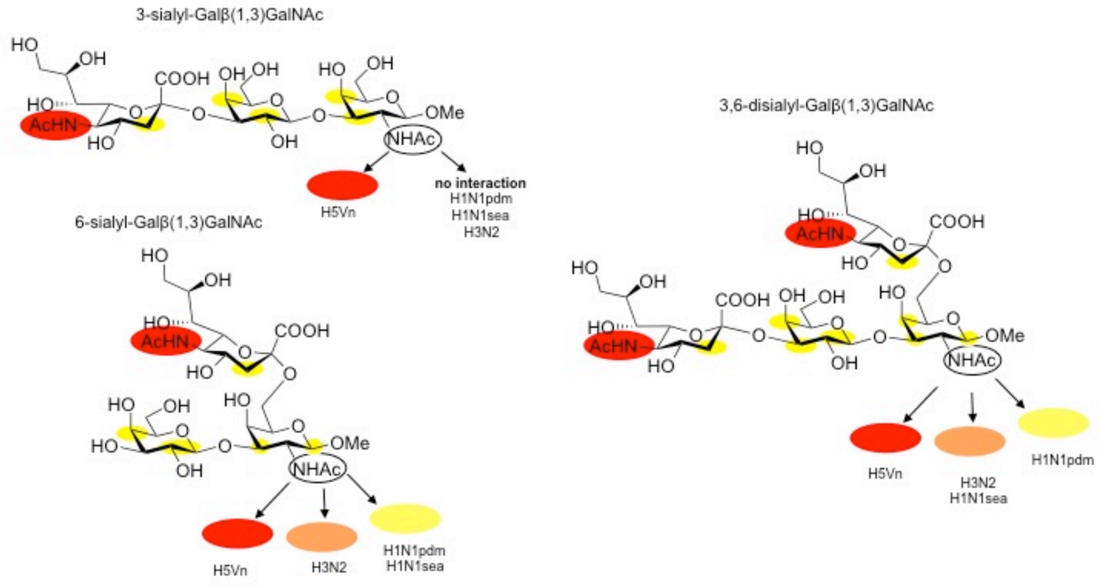

Supplementary Figure 7: Proposed binding epitope map of sialylated-Galβ1-3GalNAc.STD NMR signals of all virus strains used in this study were combined (see Supplementary Figures 4 - 6 for individual STD NMR spectra). The following colour coding was used to quantify the STD NMR effects: Strong (red), Medium (orange), Weak (yellow). Weak STD NMR signal intensities (yellow) were detected for the ring protons of Neu5Ac, Gal and GalNAc residues including $H3_{eq}$(Neu5Ac), $H3_{ax}$(Neu5Ac), H1(Gal), H2(Gal), H3(Gal), H4(Gal), H3(GalNAc) and H4(GalNAc). The strongest STD NMR signals (red) were identified for the methyl protons of the acetamido moieties of Neu5Ac and GalNAc.

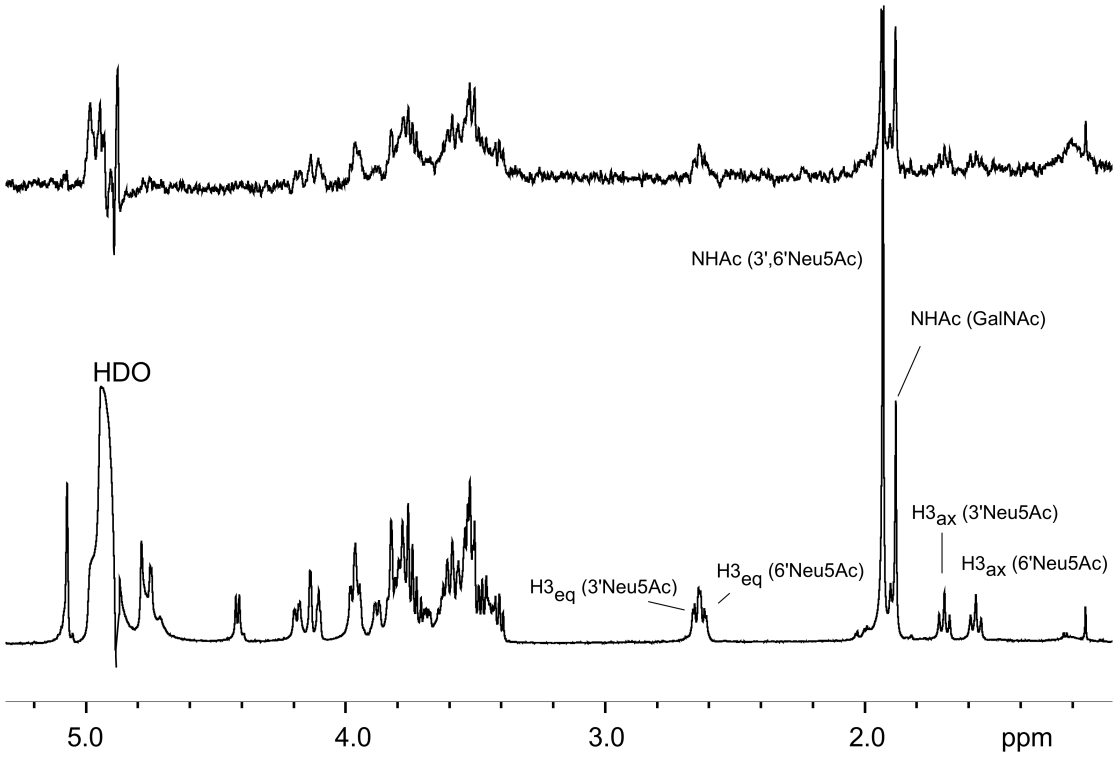

Supplementary Figure 8: ^{1}H NMR (a) and STD NMR (b) spectra of 3,6-disialyl-Galβ1-3GalNAc-1-α-serine (**6**) in complex with H1N1pdm influenza virus particles.

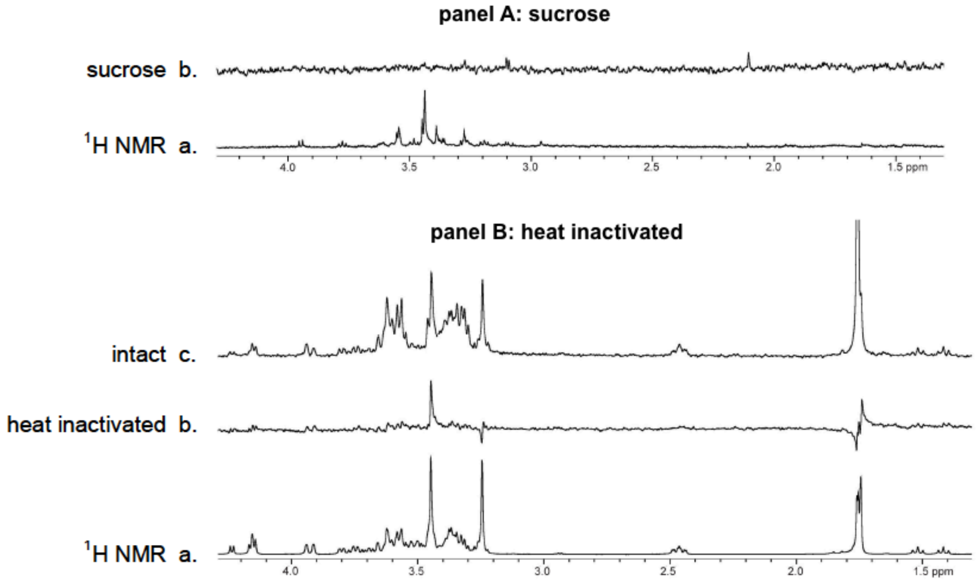

Supplementary Figure 9: Series of STD NMR control experiments to confirm the authenticity of the STD signals. **Panel A:** ^{1}H NMR (a) and STD NMR (b) spectra of an NMR sample containing H1N1pdm in the presence of 3 mM non-binding sugar sucrose. No binding of sucrose could be observed. **Panel B:** ^{1}H NMR (a) and STD NMR (b) spectra of a heat inactivated H1N1sea virus sample in the presence of an equimolar mixture of 3 mM 3-sialyl and 6-sialyl Galβ1-3GalNAc. Significantly reduced signal intensity and binding could be observed (b) compared to the virus that was intact and not heat inactivated acquired under identical experimental conditions (c). Heat inactivation of H1N1sea was achieved by incubating the virus sample for 20 min at 70 °C.

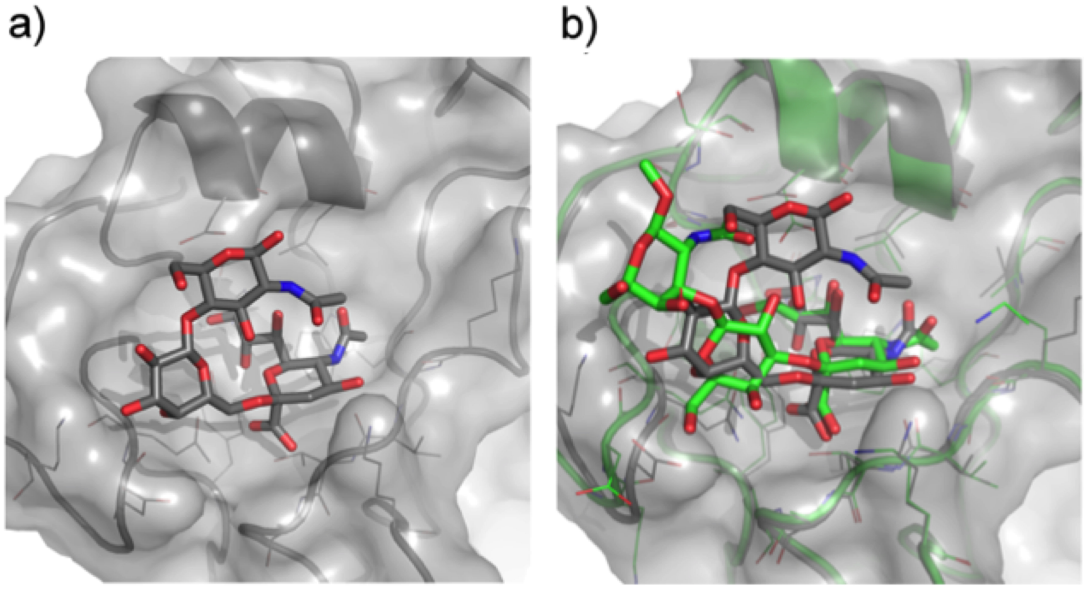

Supplementary Figure 10: a) X-Ray crystal structure of H1N1pdm in complex with 6'SLN (PDB: 3UBN) indicates a 'compact' conformation. b) Superimposition of 6'SLN (*N*-glycan, grey) with 3-sialyl-Galβ1-3GalNAc (green) further indicates similar bound conformations of their Neu5Ac and Gal residues.

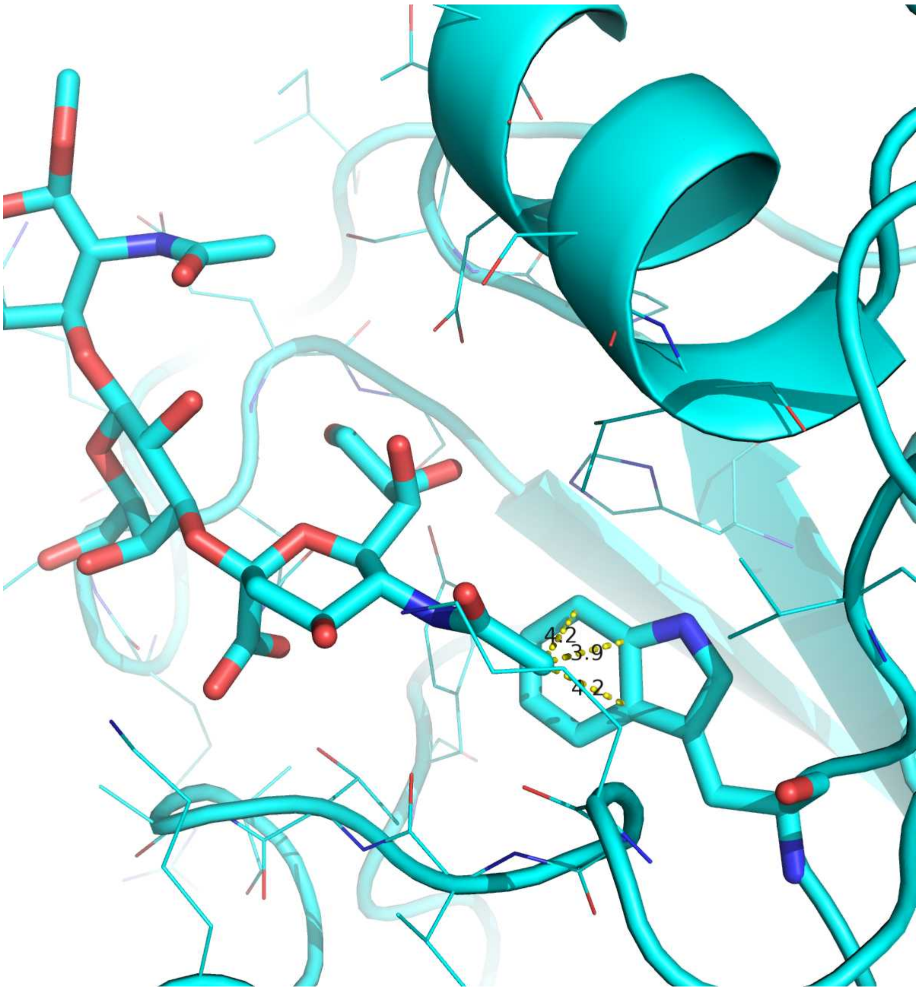

Supplementary Figure 11: MD structure of 3-sialyl-Galβ1-3GalNAc bound to H1N1pdm showing the strong hydrophobic interaction of the Neu5Ac residue with Trp-103.

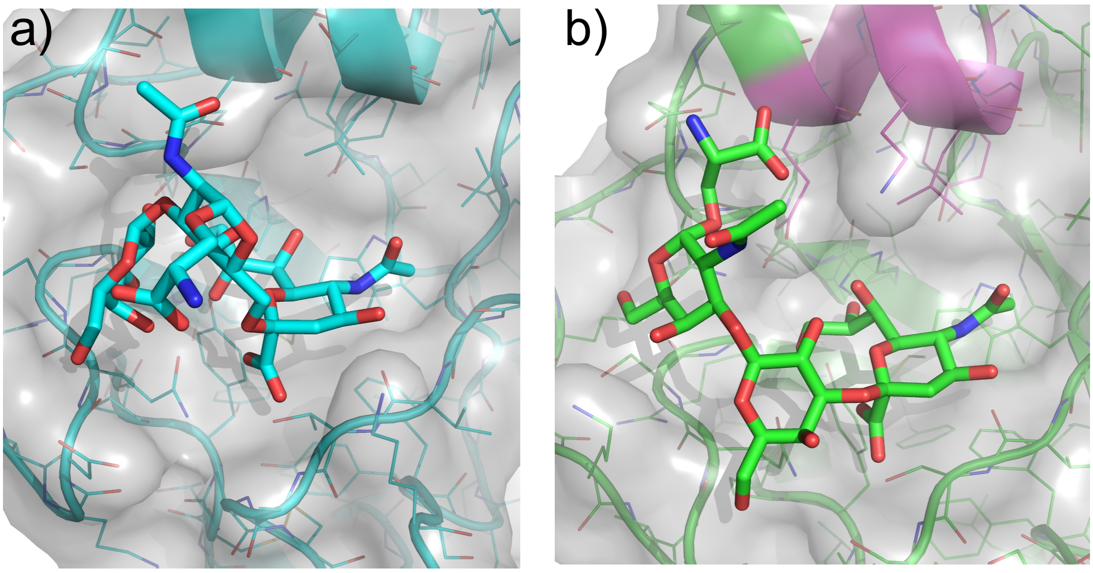

Supplementary Figure 12: a) 6-sialyl-Galβ1-3GalNAc-1-α-serine docked into H1N1pdm and b) 3-sialyl-Galβ1-3GalNAc-1-α-serine docked into H5N1. Both structures reveal a similar orientation of the sialylated *O*-glycan compared to the β-OMe aglycon.

```
H5N1     1 MEKI L FA VSL --KSDQIC GYHANNSTEQVDT ME NVTVTHAQ I E KHNG  C
H7N3     1 MNTQILALIACMLIGAK DKICLGHHAVANGTKVNTLTERG EVVNATETVETANTK IC
H7N7     1 MNTQILVFALVASIPTNADKICLGHHAVSNGTKVNTLTERGVEVVNATETVERTN P IC
H7N9     1 MNTQILVFAL A IPTNADKICLGHHAVSNGTKVNTLTERGVEVVNATETVERTN P IC

H5N1    59 DLDGV P ILRDCS AGW LGNPMCDEFINVPEWSYIVEKANPVNDLCYPGDFNDYEELK
H7N3    61  -QGKRPTDLGQCGLLGTLIGPPQCDQFLEFDA-DLIIERRE-GTDVCYPGKFTNEESLR
H7N7    61 S-KGKRTVDLGQCGLLGTITGPPQCDQFLEFSA-DLIIERRE-GSDVCYPGKFVNEEALR
H7N9    61 S-KGKRTVDLGQCGLLGTITGPPQCDQFLEFSA-DLIIERRE-GSDVCYPGKFVNEEALR

H5N1   119 HLLSRINHFEKIQIIPKSSWSSHEASLGVSSACPYQGKSSFFRNVVWLIK--KNSTYPTI
H7N3   118 QILRGSGGIDKESMG--FTY-SGIRTNGATSACRRSG-SSFYAEMKWLLSNSDNAAFPQM
H7N7   118 QILRESGGIDKETMG--FTY-SGIRTNGTTSACRRSG-SSFYAEMKWLLSNTDNAAFPQM
H7N9   118 QILRESGGIDKEAMG--FTY-SGIRTNGATSACRRSG-SSFYAEMKWLLSNTDNAAFPQM

                                      **
H5N1   177 KRSYNNTNQEDLLVLWGIHHPNDAAEQTKLYQNPTTYISVGTSTLNQRLVPRIATRSKVN
H7N3   174 TKSYRNPRNKPALIIWGVHHSGSATEQTKLYGSGNKLITVGSSKYQQSFTPSPGARPQVN
H7N7   174 TKSYKNTRKDPALIIWGIHHSGSTTEQTKLYGSGNKLITVGSSNYQQSFVPSPGARPQVN
H7N9   174 TKSYKNTRKSPALIVWGIHHSVSTAEQTKLYGSGNKLVTVGSSNYQQSFVPSPGARPQVN

H5N1   237 GQSGRMEFFWTILKPNDAINFESNGNFIAPEYAYKIVKKGDSTIMKSELEY-GNCNTKCQ
H7N3   234 GQSGRIDFHWLLLDPNDTVTFTFNGAFIAPDRASF--FRGESIGVQSDVPLDSGCEGDCF
H7N7   234 GQSGRIDFHWLILNPNDTVTFSFNGAFIAPDRASF--LRGKSMGIQSEVQVDANCEGDCY
H7N9   234 GLSGRIDFHWLMLNPNDTVTFSFNGAFIAPDRASF--LRGKSMGIQSGVQVDANCEGDCY

H5N1   296 TPMGAINSSMPFHNIHPLTIGECPKYVKSNRLVLATGLRNSPQRERR-----RKKRGLFG
H7N3   292 HNGGTIVSSLPFQNINPRTVGKCPRYVKQTSLLLATGMRNVPENPKDRKSRHRRTRGLFG
H7N7   292 HSGGTIISNLPFQNINSRAVGKCPRYVKQESLLLATGMKNVPEIPKR------RRRGLFG
H7N9   292 HSGGTIISNLPFQNIDSRAVGKCPRYVKQRSLLLATGMKNVPEIPK--------GRGLFG

H5N1   351 AIAGFIEGGWQGMVDGWYGYHHSNEQGSGYAADKESTQKAIDGVTNKVNSIIDKMNTQFE
H7N3   352 AIAGFIENGWEGLIDGWYGFRHQNAQGEGTAADYKSTQSAIDQITGKLNRLIDKTNQQFE
H7N7   346 AIAGFIENGWEGLIDGWYGFRHQNAQGEGTAADYKSTQSAIDQITGKLNRLIEKTNQQFE
H7N9   344 AIAGFIENGWEGLIDGWYGFRHQNAQGEGTAADYKSTQSAIDQITGKLNRLIEKTNQQFE

H5N1   411 AVGREFNNLERRIENLNKKMEDGFLDVWTYNAELLVLMENERTLDFHDSNVKNLYDKVRL
H7N3   412 LIDNEFSEIEQQIGNVINWTRDSMTEVWSYNAELLVAMENQHTIDLADSEMNKLYERVRK
H7N7   406 LIDNEFTEVERQIGNVINWTRDSMTEVWSYNAELLVAMENQHTIDLADSEMNKLYERVKR
H7N9   404 LIDNEFNEVEKQIGNVINWTRDSITEVWSYNAELLVAMENQHTIDLADSEMDKLYERVKR

H5N1   471 QLRDNAKELGNGCFEFYHKCDNECMESVRNGTYDYPQYSEEARLKREEISGVKLESIGIY
H7N3   472 QLRENAEEDGTGCFEIFHKCDDQCMESIRNNTYDHTQYRAESLQNRIQIDPVKLSS-GYK
H7N7   466 QLRENAEEDGTGCFEIFHKCDDDCMASIRNNTYDHSKYREEAIQNRIQIDPVKLSS-GYK
H7N9   464 QLRENAEEDGTGCFEIFHKCDDDCMASIRNNTYDHSKYREEAMQNRIQIDPVKLSS-GYK

H5N1   531 QILSIYSTVASSLALAIMVAGLSLWMCSNGSLQCRICI
H7N3   531 DIILWFSFGASCFLLLAIAMGLVFICIKNGNMRCTICI
H7N7   525 DVILWFSFGASCFILLAIAMGLVFICVKNGNMRCTICI
H7N9   523 DVILWFSFGASCFILLAIVMGLVFICVKNGNMRCTICI
```

Supplementary Figure 13: HA sequence alignment of several highly pathogenic avian influenza (HPAI) strains. The two essential amino acid residues for GalNAc binding Lys-193 and Leu-194 (H3 numbering) within the **EQTKLY**-motif (red) are labelled (*). H5N1 A/Vietnam/1203/2004; H7N3 A/chicken/Jalisco/CPA1/2012; H7N7 A/NL/219/2003; H7N9 A/Anhui/1/2013.

SYNTHESIS

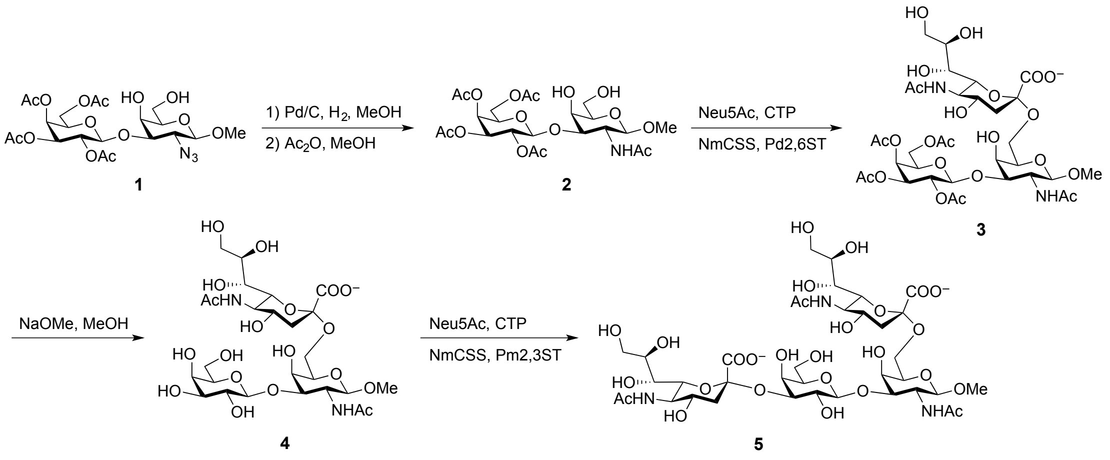

Methyl (2,3,4,6-tetra-*O*-acetyl-β-D-galactopyranosyl)-(1→3)-2-acetamido-2-deoxy-β-D-galactopyranoside (**2**) – Compound **1** was synthesised as describe in the in the literature (1). Compound **1** (1.4 g, 2.55 mmol) was dissolved in methanol (20 mL) and 10% Pd/C (140 mg) was added under the protection of argon. The mixture was hydrogenated placed in a Parr hydrogenator (H_2 45 psi) and agitated for 1 h at RT. Completion of the reaction was monitored with TLC. The hydrogenation vessel was evacuated and filled with nitrogen, the reaction mixture was filtered, acetic anhydride (1 mL) was added to the filtrate, and the solution was stirred overnight at RT. The solvent was removed under reduced pressure and the crude product was purified by chromatography on silica gel (1:2 to 1:4 hexane-acetone, v/v) to give compound **2** (0.82 g, 57% yield) as a white solid.

^{1}H-NMR (300 MHz, D_2O): δ 5.49 (d, 1 H, *J* = 3 Hz), 5.24 (dd, 1 H, *J* = 10.2 and 3 Hz), 5.14 (dd, 1 H, *J* = 10.2 and 7.8 Hz), 4.95 (d, 1 H, *J* = 8.1 Hz), 4.43 (d, 1 H, *J* = 8.1 Hz), 4.32-4.23 (m, 3 H), 4.14 (d, 1 H, *J* = 3 Hz), 4.02 (dd, 1 H, *J* = 10.5 and 8.4 Hz), 3.92-3.70 (m, 4 H), 3.54 (s, 3 H), 2.28 (s, 3 H), 2.15 (s, 3 H), 2.13 (s, 3 H), 2.05 (s, 3 H), 2.03 (s, 3 H). ^{13}C-NMR (75 MHz, D_2O): δ 174.1, 173.4, 173.1, 172.7, 172.6, 102.2, 101.4, 80.1, 74.6, 71.2, 70.6, 69.4, 68.1, 67.7, 61.9, 60.8, 57.0, 50.7, 22.3, 20.3, 20.2, 20.0, 19.9.

Methyl (2,3,4,6-tetra-*O*-acetyl-β-D-galactopyranosyl)-(1→3)-[*N*-acetyl-α-D-neuraminyl-(2→6)]-2-acetamido-2-deoxy-β-D-galactopyranoside (**3**) – Compound **2** (124.5 mg, 0.22 mmol), *N*-acetylneuraminic acid (80.4 mg, 0.26 mmol), cytidine-5'-triphosphate disodium salt (139.2 mg, 0.26 mmol) and magnesium chloride hexahydrate (89.3 mg, 0.44 mmol) were dissolved in water (11 mL). The pH was adjusted to pH 7.4-8.0, with phosphate salts at a final concentration of 0.2 M. To this solution were added the two enzymes, *N. meningitidis* CMP-sialic acid synthetase (2) (2.21 U) and *P. damsela* α2,6-sialyltransferase (2) (4.42 U). The mixture was incubated at 37 °C for 36 h, after which ice cold ethanol (22 mL) was added to quench the reaction. The reaction mixture was then centrifuged at 8000 rpm for 45 min to allow any precipitation. The top clear solution was decanted to a flask, concentrated and subject to gel filtration (Bio-gel P-2, 19mm x 120cm), eluting with water. The crude product from gel filtration was collected and further purified by chromatography on silica gel (2:1:0.1 ethyl acetate-methanol-water, v/v/v) to give compound **3** (101 mg, 52% yield) as a white solid.

^{1}H-NMR (600 MHz, D_2O): δ 5.43 (d, 1 H, *J* = 3.6 Hz), 5.19 (dd, 1 H, *J* = 10.5 and 3.3 Hz), 5.09 (dd, 1 H, *J* = 10.2 and 7.8 Hz), 4.89 (d, 1 H, *J* = 8.4 Hz), 4.37 (d, 1 H, *J* = 9.0 Hz), 4.26-4.19 (m, 3 H), 4.09 (d, 1 H, *J* = 3 Hz), 3.99-3.95 (m, 2 H), 3.87-3.75 (m, 5 H), 3.70-3.56 (m, 5 H), 3.49 (s, 3 H), 2.71 (dd, 1 H, *J* = 12 and 4.8 Hz), 2.20 (s, 3 H), 2.09 (s, 3 H), 2.08 (s, 3 H), 2.02 (s, 3 H), 2.01 (s, 3 H), 1.99 (s, 3 H), 1.67 (app.t, 1 H, *J* = 12 Hz). ^{13}C-NMR (150 MHz, D_2O): δ 177.5, 176.7, 176.0, 175.8, 175.7, 175.2, 104.7, 103.7, 103.0, 82.4, 75.6, 75.1, 74.2, 73.8, 73.0, 71.8, 70.7, 70.5, 70.2, 66.2, 65.1, 64.2, 59.7, 54.3, 53.1, 42.7, 24.7, 24.5, 22.8, 22.7, 22.5. HRMS (ESI): *m/z* calculated for $C_{34}H_{51}N_2O_{23}^-$ (M-Na)$^-$ 855.2888, found 855.2874.

Methyl β-D-galactopyranosyl-(1→3)-[*N*-acetyl-α-D-neuraminyl-(2→6)]-2-acetamido-2- deoxy-β-D-galactopyranoside (**4**) – Compound **3** (24 mg, 0.027

mmol) was dissolved in methanol (5 mL) and sodium methoxide (1 M solution in methanol, 0.1 mL) was added. The mixture was stirred for 0.5 h at RT until all starting material was consumed, producing a single product. IR-120 H^+ resin (0.5 g) was added to neutralize the reaction mixture. The reaction mixture was filtered and the filtrate was evaporated to dryness under reduced pressure to give compound **4** as a white solid.

^{1}H-NMR (600 MHz, D_2O): δ 4.41 (d, 1 H, *J* = 8.4 Hz), 4.40 (d, 1 H, *J* = 7.8 Hz), 4.18 (d, 1 H, *J* = 3 Hz), 3.98 (dd, 1 H, *J* = 10.2 and 8.4 Hz), 3.93 (dd, 1 H, *J* = 10.2 and 7.8 Hz), 3.88-3.55 (m, 16 H), 3.49 (s, 3 H), 2.70 (dd, 1 H, *J* = 12.6 and 4.8 Hz), 2.01 (s, 3 H), 1.99 (s, 3 H), 1.66 (app.t, 1 H, *J* = 12.6 Hz). ^{13}C-NMR (150 MHz, D_2O): δ 175.0, 174.8, 104.9, 102.2, 100.4, 80.0, 74.9, 73.1, 72.6, 72.5, 71.7, 70.5, 68.5, 68.2, 67.9, 63.3, 62.6, 60.9, 57.1, 51.8, 50.9, 40.2, 22.2, 22.0. HRMS (ESI): *m/z* calculated for $C_{26}H_{43}N_2O_{19}^-$ $(M\text{-}Na)^-$ 687.2466, found 687.2483.

Methyl *N*-acetyl-α-D-neuraminyl-(2→3)-β-D-galactopyranosyl-(1→3)-[*N*-acetyl-α-D-neuraminyl-(2→6)]-2-acetamido-2-deoxy-β-D-galactopyranoside (5) – Compound **4** (51.5 mg, 0.073 mmol), *N*-acetylneuraminic acid (26.9 mg, 0.087 mmol), cytidine-5'-triphosphate disodium salt (45.9 mg, 0.087 mmol) and magnesium chloride hexahydrate (29.4 mg, 0.145 mmol) were dissolved in water (5.8 mL). A pH 8.5 Tris-HCl buffer (1 M, 0.73 mL) was then added to bring the pH of the solution to 8.0-8.5. To this solution were added the two enzymes, *N. meningitidis* CMP-sialic acid synthetase (0.73 U) and *P. multocida* α2,3-sialyltransferase (3)(0.29 U). The mixture was incubated at 37 °C for 6 h, after which ice cold ethanol (7.3 mL) was added to quench the reaction. The reaction mixture was then centrifuged at 8000 rpm for 45 min to allow any precipitation. The top clear solution was decanted to a flask, concentrated and then subject to gel filtration (Bio-gel P-2, 19mm x 120cm), eluting with water. The crude product from gel filtration was collected and further purified by

chromatography on silica gel (2:1:0.4 ethyl acetate-methanol-water, v/v/v) to give compound **5** (48 mg, 65%) as a white solid. Compound **4** (14 mg, 27%) was also recovered.

^{1}H-NMR (600 MHz, D_2O): δ 4.47 (d, 1 H, J = 7.8 Hz), 4.41 (d, 1 H, J = 8.4 Hz), 4.17 (d, 1 H, J = 3 Hz), 4.04 (dd, 1 H, J = 9.6 and 3 Hz), 3.98 (dd, 1 H, J = 10.2 and 8.4 Hz), 3.93 (dd, 1 H, J = 10.2 and 7.8 Hz), 3.90 (d, 1 H, J = 3.6 Hz), 3.87-3.50 (m, 21 H), 3.49 (s, 3 H), 2.73 (dd, 1 H, J = 12.6 and 4.8 Hz), 2.70 (dd, 1 H, J = 12.6 and 4.8 Hz), 2.01 (s, 3 H), 2.00 (s, 3 H), 1.99 (s, 3 H), 1.76 (app.t, 1 H, J = 12.6 Hz), 1.66 (app.t, 1 H, J = 12.6 Hz). ^{13}C-NMR (150 MHz, D_2O): δ 175.0, 174.9, 174.8, 173.9, 173.4, 104.6, 102.2, 100.4, 99.6, 80.2, 75.5, 74.7, 73.1, 72.7, 72.6, 71.8, 71.7, 68.9, 68.4, 68.2, 68.0, 67.8, 67.3, 63.4, 62.6, 62.4, 60.9, 57.1, 51.8, 50.8, 40.2, 39.6, 22.2, 22.0. HRMS (ESI): *m/z* calculated for $C_{37}H_{60}N_3O_{27}^-$ (M-2Na+H)$^-$ 978.3420, found 978.3409.

Supplementary Information References

1. Paulsen H, Peters S, Bielfeldt T, Meldal M, & Bock K (1995) Synthesis of the Glycosyl Amino-Acids N-Alpha-Fmoc-Ser[Ac-4-Beta-D-Galp-(1-]3)-Ac-2-Alpha-D-Galn(3)P]-Opfp and N-Alpha-Fmoc-Thr[Ac-4-Beta-D-Galp-(1-]3)-Ac-2-Alpha-D-Galn(3)P]-Opfp and the Application in the Solid-Phase Peptide-Synthesis of Multiply Glycosylated Mucin Peptides with T-N and T-Antigenic Structures. *Carbohydrate research* 268(1):17-34.
2. Yu H*, et al.* (2006) Highly efficient chemoenzymatic synthesis of naturally occurring and non-natural alpha-2,6-linked sialosides: A P. damsela alpha-2,6-sialyltransferase with extremely flexible donor-substrate specificity. *Angew Chem Int Edit* 45(24):3938-3944.
3. Yu H*, et al.* (2005) A multifunctional Pasteurella multocida sialyltransferase: A powerful tool for the synthesis of sialoside libraries. *Journal of the American Chemical Society* 127(50):17618-17619.
